# Combined nature and human selections reshaped peach fruit metabolome

**DOI:** 10.1186/s13059-022-02719-6

**Published:** 2022-07-04

**Authors:** Ke Cao, Bin Wang, Weichao Fang, Gengrui Zhu, Changwen Chen, Xinwei Wang, Yong Li, Jinlong Wu, Tang Tang, Zhangjun Fei, Jie Luo, Lirong Wang

**Affiliations:** 1 The Key Laboratory of Genetic Resource Evaluation and Application of Horticultural Crops (Fruit), Ministry of Agriculture, Zhengzhou Fruit Research Institute, Chinese Academy of Agricultural Sciences, Zhengzhou, 450009 China; 2 Wuhan Metware Biotechnology Co., Ltd., Wuhan, China; 3 Boyce Thompson Institute, Cornell University, Ithaca, NY 14853 USA; 4 U.S. Department of Agriculture–Agricultural Research Service, Robert W. Holley Center for Agriculture and Health, Ithaca, NY 14853 USA; 5 Sanya Nanfan Research Institute of Hainan University, Hainan Yazhou Bay Seed Laboratory, Sanya, 572025 China; 6 College of Tropical Crops, Hainan University, Haikou, 570228 Hainan China; 7 National Horticulture Germplasm Resources Center, Zhengzhou Fruit Research Institute, Chinese Academy of Agricultural Sciences, Zhengzhou, 450009 China

**Keywords:** Peach, Multi-omics, Environmental adaptation, Nature and human selections

## Abstract

**Background:**

Plant metabolites reshaped by nature and human beings are crucial for both their lives and human health. However, which metabolites respond most strongly to selection pressure at different evolutionary stages and what roles they undertake on perennial fruit crops such as peach remain unclear.

**Results:**

Here, we report 18,052 significant locus-trait associations, 12,691 expression-metabolite correlations, and 294,676 expression quantitative trait loci (eQTLs) for peach. Our results indicate that amino acids accumulated in landraces may be involved in the environmental adaptation of peaches by responding to low temperature and drought. Moreover, the contents of flavonoids, the major nutrients in fruits, have kept decreasing accompanied by the reduced bitter flavor during both domestication and improvement stages. However, citric acid, under the selection of breeders’ and consumers’ preference for flavor, shows significantly different levels between eastern and western varieties. This correlates with differences in activity against cancer cells in vitro in fruit from these two regions. Based on the identified key genes regulating flavonoid and acid contents, we propose that more precise and targeted breeding technologies should be designed to improve peach varieties with rich functional contents because of the linkage of genes related to bitterness and acid taste, antioxidant and potential anti-cancer activity that are all located at the top of chromosome 5.

**Conclusions:**

This study provides powerful data for future improvement of peach flavor, nutrition, and resistance in future and expands our understanding of the effects of natural and artificial selection on metabolites.

**Supplementary Information:**

The online version contains supplementary material available at 10.1186/s13059-022-02719-6.

## Background

Peach (*Prunus persica*) is among the most widely consumed fruit crops in the world. Its production ranks the fourth after apple, pear, and grape, contributing to a $4.6 billion industry annually (https://www.fao.org/faostat/). Peach fruit and its products provide many of the essential and beneficial nutrients in the human diet. Consumption of fruits and vegetables has been associated with reduced risk of some chronic diseases such as cardiovascular disease and cancer [1, 2]. It is well known that flavor and nutritional values of food crops are ultimately determined by their chemical compositions [3]. Tracking metabolic patterns during domestication and improvement is an important approach to explore the interaction among nature, human, and plants. For example, wheat domestication was first characterized by a reduction in unsaturated fatty acids and then altered amino acid content at different stages [4]. Different with field crops, the domestication of fruit crops tend to reduce bitterness [5] and acidity [6], while increase sweetness [7] and attractive color [8]. However, how artificial selection has reshaped the metabolite profiles of fruit crops remains largely unknown.

As sessile beings, plants have evolved a unique and sophisticated response to environmental stresses through regulating metabolism to escape from adverse conditions [9]. In rice, the accumulation of low temperature upregulates the enzymes involved in starch degradation, sucrose metabolism, and the glyoxylate cycle and enhances abscisic acid (ABA) signaling while represses cytokinin signaling [10]. Increased ABA level has also been found in response to water-deficit stress through affecting the accumulation of various amino acids and sugars [11]. In peaches, some reports have documented the change of metabolite levels in fruits under cold storage and UV-B irradiation [12, 13]. However, effects of external environments and human selections on metabolites are poorly understood.

Peach has served as a model species for genomic research of Rosaceae, making a comprehensive metabolomic study of this species imperative. Metabolic quantitative trait loci (mQTLs) have been identified in peach based on linkage maps using the SNP array [14, 15]. However, the underlying genes remain elusive because of the relatively low resolution of the genetic maps. In recent years, genome-wide association studies coupled with targeted metabolome analysis (or mGWAS) make it possible to simultaneously screen a large number of accessions to understand the genetic basis of metabolic diversity and their relevance to complex traits. Such studies have been carried out in some important crops and model plant species, including rice, tomato, Arabidopsis, and maize [16–19]. Results from these studies provide an essential reference to help understand the genetic basis of natural variation of the metabolomes and to facilitate the breeding of elite varieties with increased resistance to detrimental stresses and enhanced nutritional values.

In this study, through comprehensive metabolic profiling, mGWAS, and expression quantitative trait loci (eQTL) analyses, we provide the genetic basis of metabolite changes in the process of peach evolution. A total of 28 and 32 hotspots in the peach genome that regulate the metabolite variations in 252 diverse peach accessions were identified in two seasons, 2015 and 2016, respectively. We discovered several novel metabolites that are involved in peach adaptation to different environments. In addition, we identified footprints of artificial selection associated with fruit flavor and nutrition. This study demonstrates that the high-resolution mapping of a large number of metabolites might significantly improve the efficiency of important gene identification and pathway elucidation, providing insights into genetic and biochemical basis of peach fruit metabolomes and also valuable resources for crop improvement through metabolomics-assisted breeding.

## Results

### Metabolic profiling of peach fruits

**Fig. 1:**
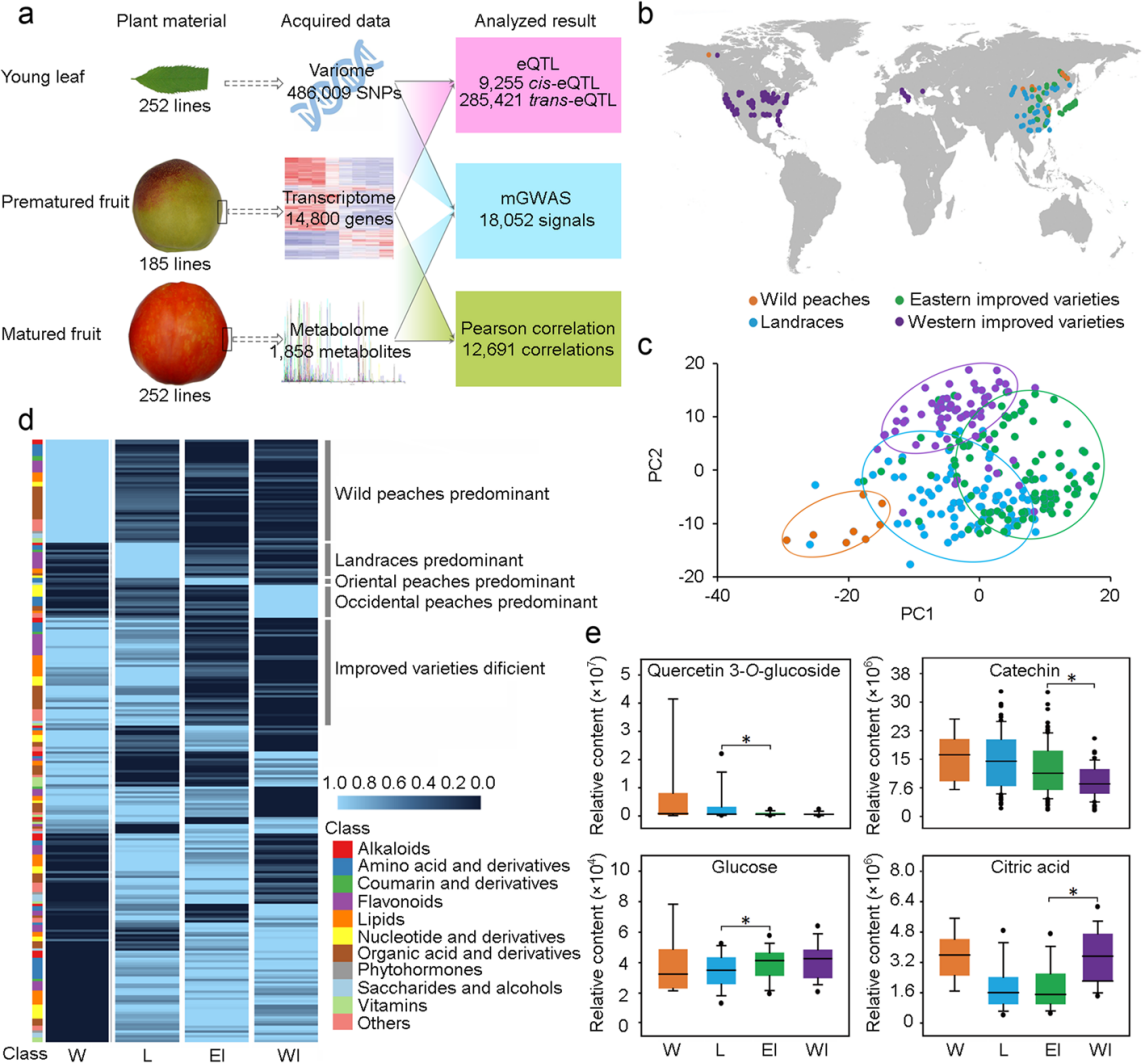
Metabolome profiles of peach accessions. **a** Summary of the experimental pipelines and data presented in this study. **b** Geographic distribution of peach accessions sampled in this study. Wild peaches, landraces, eastern improved varieties, and western improved varieties are indicated by pink, blue, green, and purple circles, respectively. **c** Principal component analysis of 252 peach accessions according to their metabolome profiles. **d** Heatmap of the relative levels of all annotated metabolites in four peach populations, wild (W), landraces (L), eastern improved varieties (EI), and western improved varieties (WI). Different classes of metabolites are plotted in the left column and shown in different colors. **e** Boxplots of representative metabolites in W, L, EI, and WI groups

The overall experimental design and the data obtained in this study are shown in Fig. 1a. Firstly, to determine the metabolic profiles of peach fruits, mature fruits harvested in two seasons (2015 and 2016) from a diverse panel comprised of 252 accessions (Fig. 1b; Additional file 2: Table S1) were analyzed using a targeted high-throughput LC-MS/MS approach, which detected a total of 1858 distinct metabolite features (Additional file 3: Table S2). Of the detected metabolites, 257 could be annotated. We found that 33.4% and 27.5% of the metabolites displayed broad-sense heritability greater than 0.5 in 2015 and 2016, respectively (Additional file 1: Fig. S1a), and 71.04 and 71.83% of the metabolites had the coefficient of variations (CVs) greater than 50% (Additional file 1: Fig. S1b), suggesting great variations of metabolites in different peach fruits. Flavonoids in fruit showed the highest CVs with an average of 207.91%, ranging from 25.02% (Quercetin 4’-*O*-glucoside) to 731.01% (rutin), while saccharides and alcohols showed the lowest CVs with an average of 46.35%. Among the 257 annotated substances, 232 showed significant (*r* > 0.18 and *p* ≤ 0.01) (Additional file 1: Fig. S2a) and 89 showed extremely significant (*r* > 0.62, Additional file 1: Fig. S2b) correlations between seasons 2015 and 2016, suggesting a stable repeatability.

Based on the levels of all detected metabolites, principal components analysis (PCA) was performed (Fig. 1c), which largely separated the accessions into four distinct clusters: wild and ornamental peaches (W), landraces (L), and improved varieties (I) that can be further categorized into eastern (EI) and western (WI) improved varieties, indicating dynamic changes of metabolite profiles during the evolution from wild peaches to improved varieties. This is consistent with our previous studies based on SNPs identified from genome resequencing [20, 21].

To reveal a possible reshaping of the peach metabolome during evolution, the specific annotated metabolic components in a peach group were examined. The results showed that the W group had the most specific components (44), followed by L (15), WI (14), and EI (3) (Fig. 1d; Additional file 4: Table S3). We further identified differential metabolites between each of the above two peach groups (Additional file 1: Fig. S3-S5) according to FC (fold change) ≥ 1.5 or ≤ 0.67 and variable importance in the projection (VIP) ≥ 1. A total of 502 and 620 metabolites were identified when comparing wild peaches and landraces in seasons 2015 and 2016, respectively, including mainly flavonoids and organic acids and derivatives (Additional file 5: Table S4). For example, some oxyflavonoids (isorhamnetin *O*-hexoside and rutin) were decreased by 90% from wild peaches to landraces in both seasons. In contrast, 347 and 321 differed significantly between landraces and improved varieties in 2015 and 2016, respectively (Additional file 6: Table S5), and these continued to be mainly flavonoids and organic acids. Metabolites with the most significant declines in both seasons were cyanidin 3-*O*-rutinoside, pelargonidin 3-glucoside, and rutin. Using season 2016 as an example, we found that 24 metabolites have continuously decreased over the evolutionary history of peach, including mainly 11 organic acids and 8 flavonoids. The changes of some representative metabolites are shown in Fig. 1e.

Generally, among the 10 classes of annotated metabolites, flavonoids and organic acids are obviously selected during peach domestication and improvement. Our results suggest that the combined selection pressure by nature and human has continuously reshaped the metabolome of peach. In addition, a total of 22 metabolites were identified that differed between the EI and WI in both seasons (Additional file 7: Tables S6; Additional file 8: Table S7), including 12 with higher levels in EI and 10 in WI, suggesting the regional influence of human selection on fruit metabolites.

### Multi-omics analyses to dissect the genetic basis of peach metabolome

**Fig. 2:**
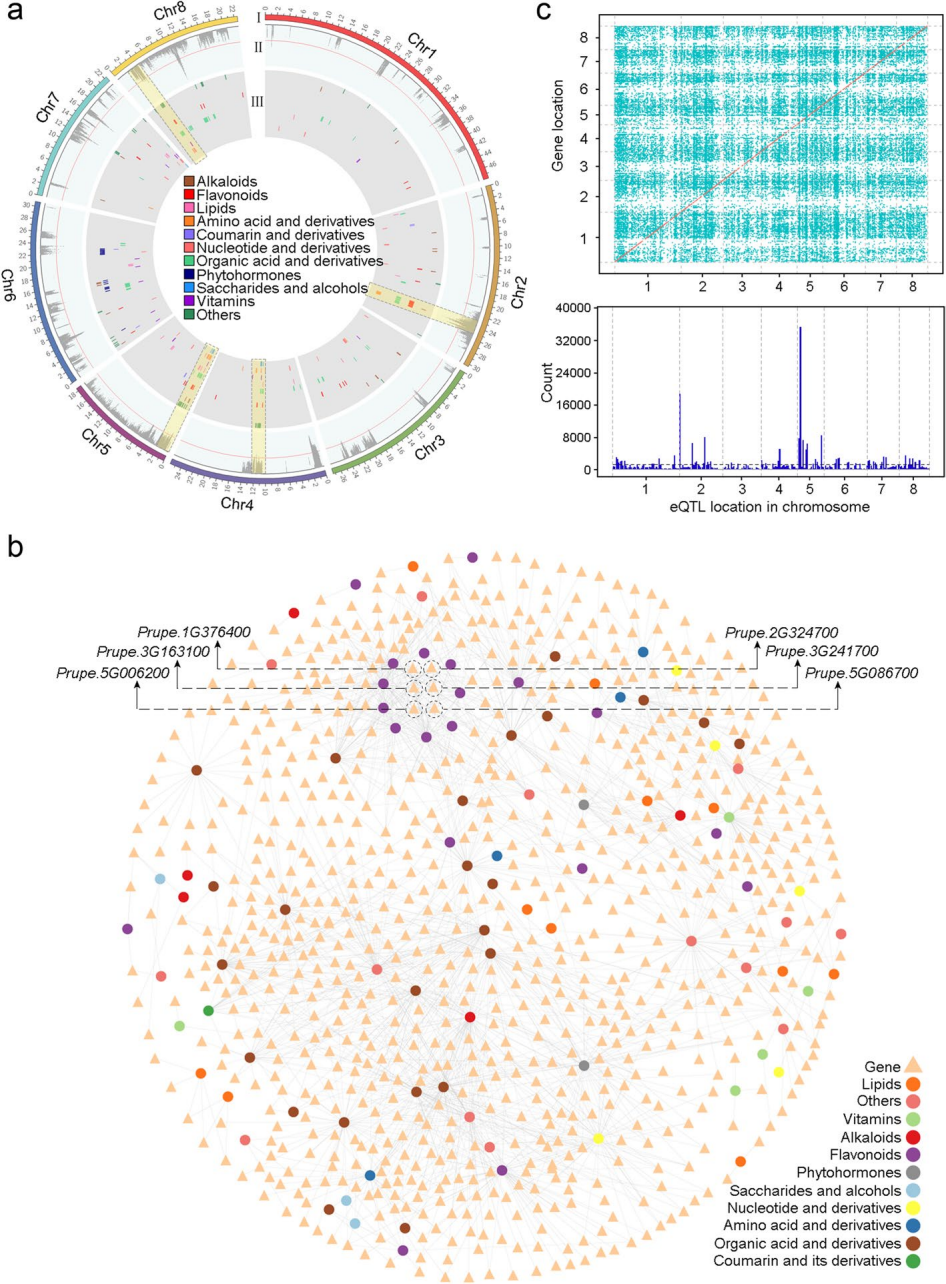
Genomic distribution of mGWAS and eQTL signals. **a** mGWAS signals of different classes of metabolites. I, II, and III indicate chromosome, unannotated metabolites, and annotated metabolites, respectively. **b** Correlation network of 94 annotated metabolites and 1144 expressed genes. **c** Genome-wide mapping of eQTLs. The upper figure indicates cis-eQTLs (brown points) and trans-eQTLs (green points) in the peach genome. The lower figure indicates the distribution of eQTL density (number of eQTLs per 1-Mb windows) along the chromosome

To reveal the molecular mechanism of metabolite changes during evolution, a total of 486,009 high-quality SNPs identified from the previous studies (Additional file 2: Table S1) [21, 22] were used for mGWAS analysis. We identified 8685 and 10,412 lead SNPs corresponding to 582 and 628 metabolites in seasons 2015 and 2016 (Table 1; Fig. 2a), respectively, with an average of 14.0% and 13.7% explained variations (*R*^2^) (Additional file 9: Table S8; Additional file 1: Fig. S6). Of these metabolites, 81.1% and 80.7% had multiple lead SNPs in 2015 and 2016, respectively, with 298 and 337 metabolites having more than five lead SNPs (Additional file 10: Table S9). Few metabolites were controlled by only one primary locus that explained over 10% of the natural variation, such as L-phenylalanine (mr262) and L-leucine (mr1325). Among all association signals, we identified 25 (threshold = 50 associated lead SNPs per Mb) and 23 (threshold = 59 associated lead SNPs per Mb) potential “hotspots” associated with metabolites in 2015 and 2016, respectively (Additional file 1: Fig. S7; Additional file 11: Table S10), including four principal hotspots located on Chr. 2: 23–26 Mb, Chr. 4: 1–2 Mb, Chr. 5: 0–3 Mb, and Chr. 8: 3–5 Mb (Fig. 2a). Enrichment analysis of the metabolites associated with the above four hotspots revealed that the target metabolites were mainly flavonoids (such as malvidin-3-*O*-glucoside, methylChrysoeriol 5-*O*-hexoside, quercetin 3-*O*-glucoside, and chrysoeriol 5-*O*-hexoside), followed by organic acids (such as syringetin *O*-hexoside, citric acid, methylcitric acid, and 3-*O*-feruloylquinic acid), amino acids (such as 3-hydroxykynurenine and histidinol), and nucleotides (such as hypoxanthine).

**Table 1:** Summary of genome-wide significant associations identified in mGWAS

	Associated lead SNPs identified in 2015	Associated lead SNPs identified in 2016	Associated lead SNPs identified in both 2015 and 2016
Number of the traits with obvious associated lead SNPs	582	628	202
Number of associated lead SNPs^a^	8685	10,412	1045
SNPs above 20% of the variation	359	656	108
Maximum explained variations	48.4%	51.5%	46.8%
Explained variation per SNP	14.0%	13.7%	15.0%

^a^SNP with the lowest *P*-value in a defined region

To assist in the identification of candidate genes underlying the natural variation of the metabolome, we collected fruits at 15 days prior to ripening of 185 accessions in 2016 for transcriptome sequencing (Additional file 12: Table S11). A total of 14,800 genes were detected using this RNA sequencing (RNA-seq) dataset (Additional file 1: Fig. S8). We found that a total of 1222 genes were differentially expressed between different peach groups (Additional file 1: Fig. S9), including 920 in the comparison of W/(L+I), 207 in L/I, and 218 in EI/WI (fold change ≥ 2 or ≤ 0.5, *p* ≤ 0.05) (Additional file 13: Table S12; Additional file 1: Fig. S10). KEGG enrichment analysis revealed that differentially expressed genes (DEGs) in W/(L+I) and EI/WI were mainly involved in indole alkaloid biosynthesis, and those in L/I were involved in flavonoid biosynthesis (Additional file 1: Fig. S11).

Correlation analysis between transcriptome and metabolome in 2016 identified a total of 12,691 expression-metabolite correlations (*r* > 0.4, *p* < 2.23 × 10^−6^) involving 564 chemicals and 1815 genes (Additional file 14: Table S13), with 204 (36.2%) of the metabolites correlated with more than ten genes and 143 (25.4%) correlated with only one gene (Additional file 1: Fig. S12). For example, a lipid substance, palmitaldehyde, was correlated with *Prupe.6G307900*, which encodes an esterase/lipase. Finally, we identified 85 candidate genes for 220 SNP loci of 77 metabolites in 2016, which were located in the flanking regions of lead SNPs and showed a high correlation (*r* > 0.4, *p* < 2.23 × 10^−6^) between their expression profiles and metabolite contents (Additional file 15: Table S14). We also conducted a weighted gene co-expression network analysis (WGCNA) and identified 47 modules of highly correlated genes (Additional file 16: Table S15; Additional file 1: Fig. S13), some of which were involved in specific pathways (Additional file 17: Table S16). For example, modules 28 enriched in flavonoid biosynthesis was also found to be related to 61 substances, including 8 annotated metabolites belonging to flavonoids (Additional file 18: Table S17).

To identify genetic variants involved in regulating gene expression, we next performed eQTL analysis using the aforementioned 486,009 SNPs (MAF > 0.02 and missing rate < 20%) and 13,050 genes (FPKM > 1 in > 80% accessions). As previously reported, the lead SNP within a 30-kb interval was selected and defined as an eQTL (Zhu et al., 2018). A total of 9255 cis-eQTLs (Additional file 19: Table S18) and 285,421 trans-eQTLs (Additional file 20: Table S19) were identified for 1792 and 1257 genes (Fig. 2c), respectively. A total of 36 trans-eQTL hotspots (Fig. 2c; Additional file 21: Table S20; threshold = 1355 trans-eQTLs per Mb) was identified at the whole genome level. A high consistency between eQTLs (Fig. 2c) and mGWAS (Additional file 1: Fig. S7) on some chromosomes was found. For example, among the 36 eQTL hotspots, 13 were overlapped with the mGWAS hotspots. The result showed an inherent connection among genomic variation, gene expression, and metabolome. The abundant resources help us build a network between the three datasets and facilitate to identify key genes of targeted metabolites and analyze their regulatory mechanisms.

### Evolution and regulation of flavonoids

**Fig. 3:**
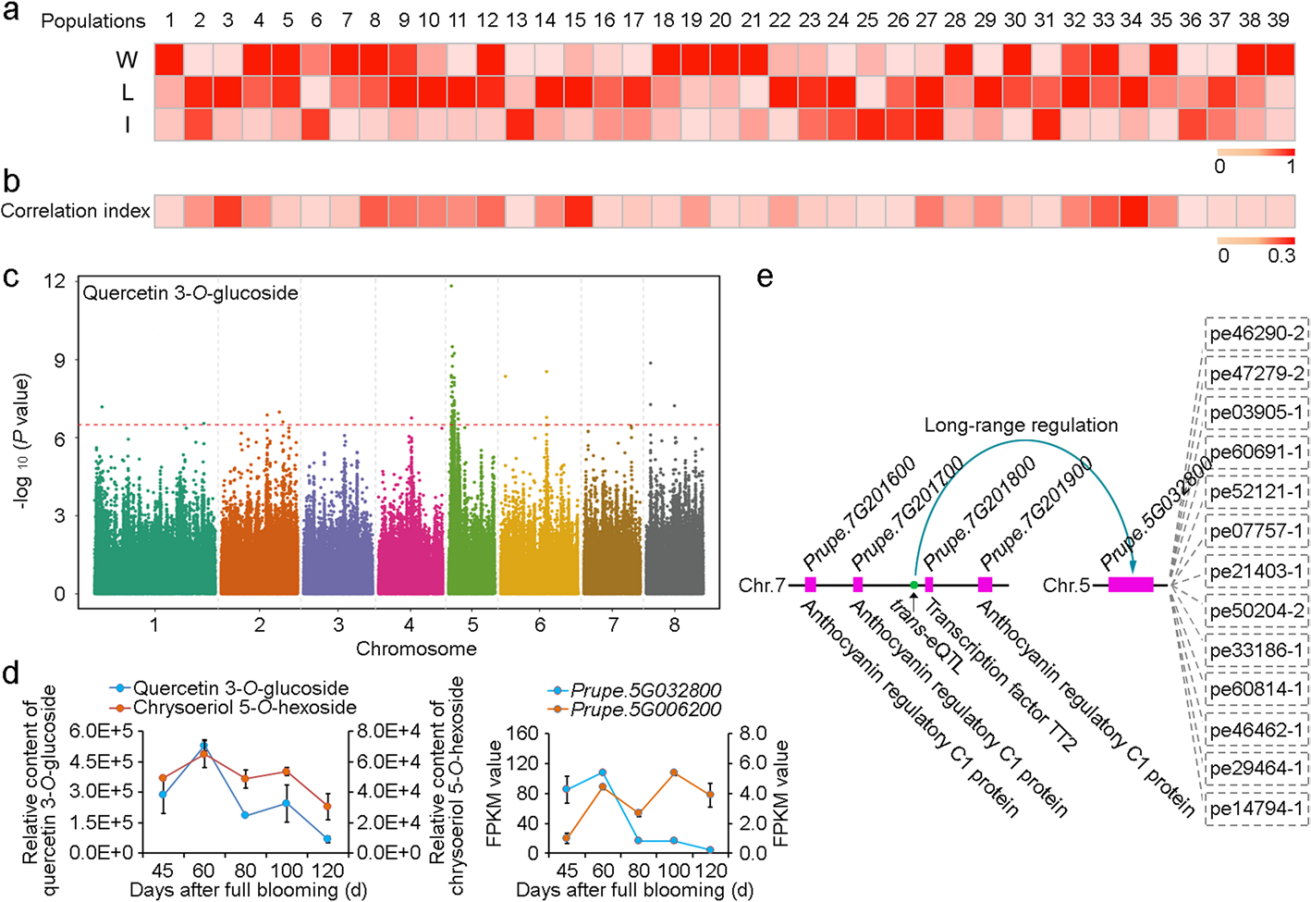
Identification of candidate genes in the lead SNP hotspots associated with flavonoids. **a** Heatmap of profiles of all flavonoids detected in this study. The relative flavonoid contents in wild peaches (W) and landraces (L) were scaled to the improved varieties (I) group for each metabolite. Numbers 1 to 39 correspond to the 1st to 39th flavonoids in Additional file 2: Table S2. **b** Correlation index between metabolite contents and their antioxidant activities in the sampling panel. **c** Manhattan plots of mGWAS of quercetin 3-*O*-glucoside in 2016. **d** Contents of chrysoeriol 5-*O*-hexoside and quercetin 3-*O*-glucoside and expression of *Prupe. 5G006200* and *Prupe.5G032800* in peach variety “Zheng Bai 5-6#” during fruit development. **e** Network between candidate genes regulated by a trans-eQTL (Chr. 7: 18,797,533 bp) and corresponding metabolites. Blue lines indicate the regulations between eQTLs (green circle on Chr. 7) and target genes (purple boxes on Chr. 5). The gray dotted lines indicate the correlations between gene and metabolites

Among all metabolites, flavonoids were the most critical because they had the largest CVs in all samples, indicating a complex function. Of the 39 annotated flavonoids, 33 had lower levels in improved varieties than in wild peaches or landraces, indicating strong negative selection for flavonoids during peach breeding (Fig. 3a). These metabolites included several bitter taste components such as catechin, prunin, and rutin.

Interestingly, flavonoids were also reported to be key nutrients in peaches [23]. To verify this relationship, we measured the browning degree of 186 of 252 accessions in 2016 to represent the antioxidant activity of their mature fruits (Additional file 22: Table S21). The result showed that among the 1858 detected metabolites, 165 were positively correlated with the antioxidant activity of peaches (Additional file 23: Table S22). Among the 33 flavonoids mentioned above, most of them had a high correlation with antioxidant activity (Fig. 3b), such as quercetin *O*-acetylhexoside, eriodictyol-7-*O*-glucoside, and quercetin 3-*O*-glucoside.

To confirm that the selection of these flavonoids are reliable, we identified 95 genomic regions related to domestication (wild and ornamental groups versus landraces), 110 related to improvement (landraces versus improved varieties), and 89 related to the differentiation between EI and WI groups (Additional file 24: Table S23). We found that 1653 of the identified lead SNPs in 2016 were located in domestication regions, 1735 in improvement regions, and 1377 in differentiation regions, accounting for 15.9, 16.7, and 13.2%, respectively, of the total identified associated signals and corresponding to 368, 358, and 313 metabolites (Additional file 25: Table S24). Among the 82 lead SNPs associated with 11 flavonoid metabolites (Additional file 9: Table S8), most were located in the principal hotspots on chromosomes 2 and 5, which were selected by improvement and differentiation, respectively (Additional file 1: Fig. S14). Lead SNPs of flavonoids related to bitterness, such as rutin, were found to be selected continuously during the process of domestication and improvement, and some nutrition-related metabolites, such as quercetin 3-*O*-glucoside, were mainly selected during the improvement stage (Additional file 1: Fig. S14).

To rewire the nutrition of improved varieties, we attempted to identify key regulatory genes of these nutritional flavonoids. Interestingly, among the abovementioned nutritional flavonoids (Additional file 23: Table S22), association signals for two major metabolites (quercetin 3-*O*-glucoside and chrysoeriol 5-*O*-hexoside) were mainly mapped on the hotspots at the top of chromosome 5 (Additional file 9: Table S8; Fig. 3c). In detail, the lead SNPs associated with chrysoeriol 5-*O*-hexoside and quercetin 3-*O*-glucoside were all located at Chr. 5: 1,129,225 bp in 2015 and 2016. Within this region (Chr. 5: 0–3 Mb), a hub gene in the correlation network (Fig. 2b), *PpBL* (*Prupe. 5G006200*), showed an obvious correlation between its expression profile and the contents of apigenin-5-*O*-glucoside, quercetin *O*-acetylhexoside, quercetin 3-*O*-glucoside, naringenin-7-*O*-glucoside, isorhamnetin *O*-hexoside, eriodictyol-7-*O*-glucoside, and chrysoeriol 5-*O*-hexoside (Additional file 14: Table S13). *PpBL* was reported to be involved in the synthesis of anthocyanins by regulating the *PpMYB10* gene [24]. However, the contents of chrysoeriol 5-*O*-hexoside and quercetin 3-*O*-glucoside had a weak correlation (*r* = 0.19 and 0.13) with the expression of *PpBL* in peach cultivar “Zheng Bai 5-6” throughout the entire fruit development process (Fig. 3d). *Prupe.5G032800*, close to the hotspots (Chr. 5: 3,729,774-3,733,907 bp), encoding a dihydroflavonol-4-reductase (DFR), which mediates the first step of the anthocyanin biosynthesis [25], was among the most likely candidates (Additional file 26: Table S25) that showed high correlations (*r* = 0.70 and 0.88) with the contents of both chrysoeriol 5-*O*-hexoside and quercetin 3-*O*-glucoside during fruit development (Fig. 3d). Meanwhile, combined with the eQTL results, we found that *Prupe.5G032800* might be regulated by a trans-eQTL (Chr. 7: 18,797,533 bp) which was located between *Prupe. 7G201700* encoding an anthocyanin regulatory C1 protein and *Prupe. 7G201800* encoding a transcription factor TT2 (Fig. 3e). These genes were reported to be involved in phenylpropanoid biosynthesis [26, 27], suggesting the potential role of the trans-eQTL in future breeding project for highly nutritional peaches.

### Amino acid contributed to the environmental adaptation of peach landraces

Growth of plants is continuously affected by all kinds of abiotic and biotic stresses, such as extreme temperatures, drought, pest, and pathogen attack [28–32]. Moreover, accumulations of secondary metabolites are most likely to be modified as crops spread into new environments or have been bred for new or improved traits [33].

To verify the speculation, the annual average temperature and rainfall in the habitats of 76 landraces were analyzed to identify 515 differential metabolites (fold change ≥ 1.5 or ≤ 0.67, VIP ≥ 1) that were associated with temperature, including 68 annotated metabolites (Additional file 27: Table S26; Additional file 1: Fig. S15) and 423 (66 annotated) associated with rainfall (Additional file 28: Table S27; Additional file 1: Fig. S16). The main classes of metabolites associated with temperature were amino acids and nucleotide metabolites, which have been reported to be involved in plant resistance to low temperature [33–35]. We next treated mature trees bearing fruits with low temperature (4 °C) for 6 days to analyze the short-term effects of cold stress on fruit metabolites (Additional file 1: Fig. S17). A total of 25 annotated metabolites were identified (fold change ≥ 1.5 or ≤ 0.67, VIP ≥ 1; Additional file 29: Table S28), also dominated by amino acids and nucleotide metabolites. Among them, four were repeatedly found in the differential substances of various geographic groups (Additional file 1: Fig. S17c), including two compounds of amino acid and derivatives (L-leucine and L-valine), one compound of nucleotide and derivatives (N^2^,N^2^-dimethylguanosine), and one alkaloid (betaine). The main classes of metabolites associated with rainfall were amino acids and derivatives, which have been reported to play a role in plant resistance to drought [31, 36–38]. We also performed wide-targeted metabolic profiling for peach plants under drought stress (Additional file 1: Fig. S18). In total, 73 annotated metabolites were found to respond to this short-term drought treatment (Additional file 30: Table S29), including flavonoids, lipids, and nucleotides. The common metabolites identified by these two methods (Additional file 1: Fig. S18c) included an amino acid and derivative (DL-homocysteine), three flavonoids (genistein, isorhamnetin-3-o-rutinoside, and nicotiflorin), an organic acid and derivative (eudesmoyl quinic acid) and one nucleotide and derivative (guanosine 3’,5’-cyclic monophosphate). Interestingly, we found that 21 metabolites responded to both the long-term low temperature and little rainfall (Additional file 31: Table S30), most of which were amino acids such as L-valine, followed by nucleotides and alkaloids such as betaine, which have been reported to be associated with stress [31, 33, 36, 38–42]. Spraying the L-valine onto the young peach trees also confirmed the function of L-valine in low temperature resistance (Additional file 1: Fig. S19).

**Fig. 4:**
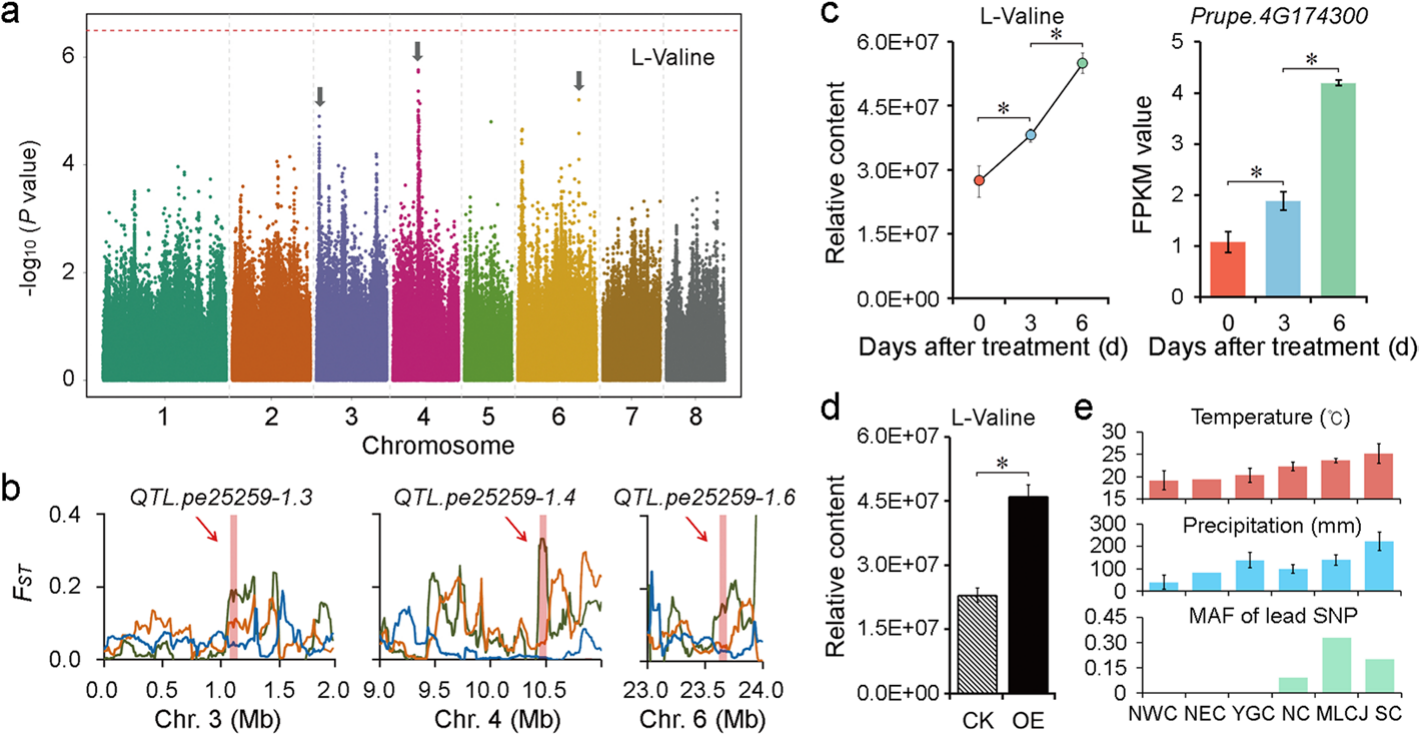
Discovery of key genes regulating L-valine contents. **a** Manhattan plots of mGWAS of L-valine in 2016. **b** Distribution of *F*_*ST*_ values related to domestication (green), improvement (orange), and differentiation (blue) on chromosomes 3, 4, and 6. Association signals detected in 2016 for L-valine are highlighted and pointed by red arrows. **c** Contents of L-valine and expression of *Prupe.4G174300* in fruits treated with low temperature (4 °C). **d** L-valine contents in peach fruits transiently overexpressing *Prupe.4G174300* (OE) and control (CK). **e** Monthly average temperature and precipitation over 30 years and minor allele frequency (MAF) of landraces belonging to different geographic groups. NWC, NEC, YGC, NC, MLCJ, and SC indicate northwest China, northeast China, Yungui plateau, northern China, the middle and lower reaches of the Yangtze River, and southern China, respectively, as described in Additional file 2: Table S1

We further analyzed the evolutionary mechanisms of the elite alleles using L-valine as an example. We found that mGWAS peak signals of L-valine were on chromosomes 3, 4, and 6 (Fig. 4a), and all these three potential association signals were under selection by domestication and improvement (Fig. 4b). We focused on the mQTL regions on chromosome 4 and found 17 of 53 expressed genes in this region showed a similar trend with the L-valine content during fruit development (Additional file 1: Fig. S20). Among the 17 genes, *Prupe.4G174300*, encoding a branched-chain amino acid aminotransferase responsible for catalyzing the conversion of 3-methyl-2-oxobutanoate to valine, was the most possible candidate for L-valine biosynthesis. Both *Prupe.4G174300* and L-valine showed similar changes under short-term cold induction (Fig. 4c). Overexpression of *Prupe.4G174300* in peach flesh resulted in a higher accumulation of L-valine (Fig. 4d), which strongly suggests *Prupe.4G174300* is involved in L-valine biosynthesis. Analyzing the changes of MAF of the lead SNP in different geographic groups again confirmed the role of this metabolite in coping with low temperature and drought stress (Fig. 4e).

### Regional selection of flavor leads to the variation of anti-cancer activity in peach

In addition to flavonoids (such as rutin), some primary metabolites related to flavor, such as malic acid, citric acid, fructose, and glucose, were also subject to human selection (Fig. 1e). However, mGWAS did not detect any association signals for glucose but identified one for sorbitol (Chr. 4: 11,212,067 bp in 2015; Additional file 9: Table S8), which is same as that reported in our previous study [21]. As sorbitol is not the main sugar component in fruits, acid content was selected to analyze the changes in peach flavor during peach breeding. Among the different acid components, metabolomics analysis indicated that the content of malic acid was significantly reduced during improvement, while citric acid presented at higher levels in improved varieties than in landraces. Further analysis showed that both malic acid and citric acid were accumulated at a much higher level in western peaches than in eastern varieties (Additional file 4: Table S3), possibly related to the preference of more acidic fruits of breeders and consumers in Europe or America.

**Fig. 5:**
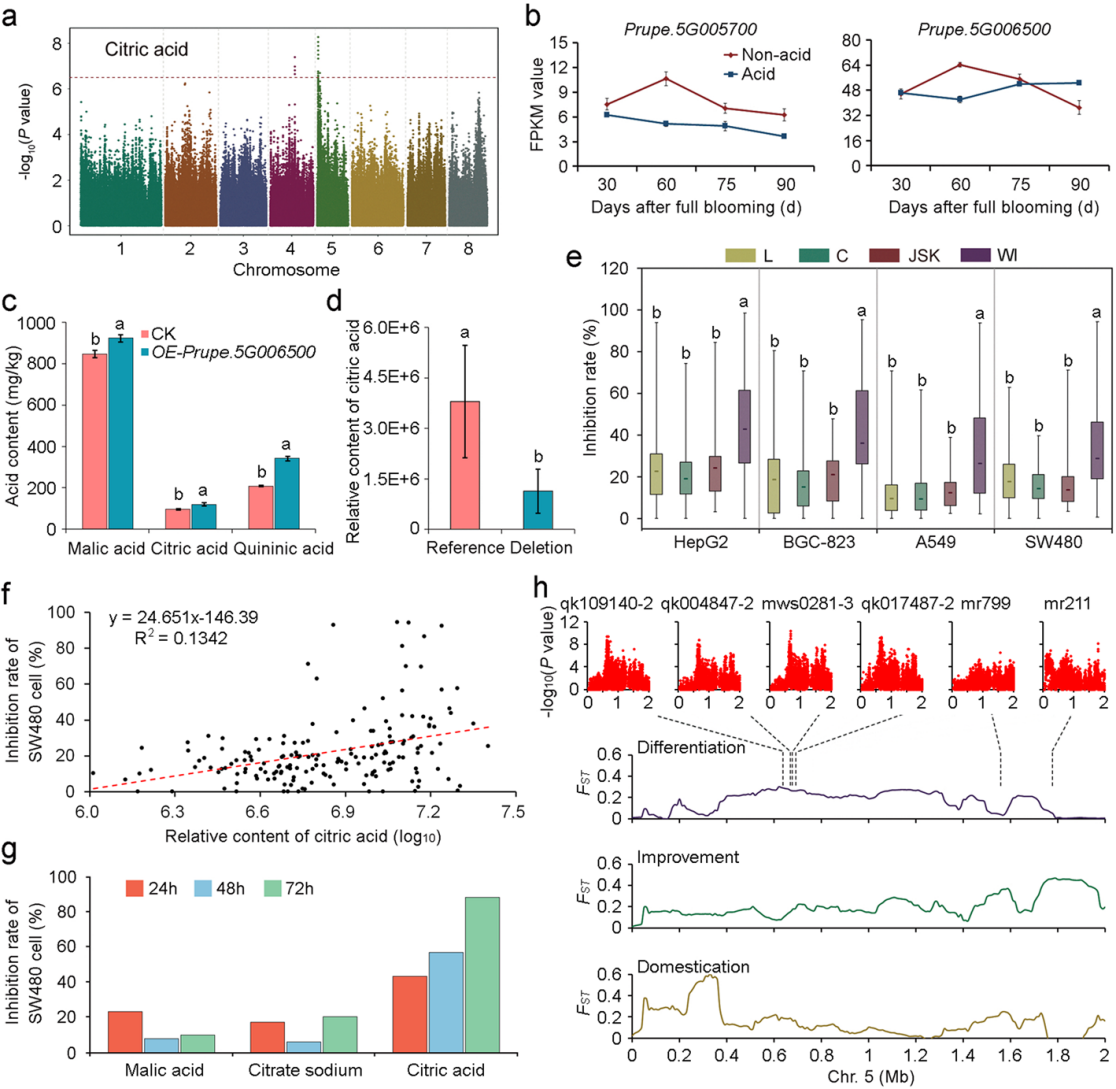
Identification of anti-cancer metabolites and their regulatory genes. **a** Manhattan plots of mGWAS for citric acid in 2016. **b** Expression of *Prupe.5G005700* and *Prupe.5G006500* during fruit development of acid and non-acid varieties. **c** Acid contents in peach fruits transiently overexpressing *Prupe.5G006500* (*OE-Prupe.5G006500*) and control (CK). Data shown are means ± SD of three biological replicates. **d** Relative contents of malic acid in accessions with different genotypes of *Prupe.5G006500*. **e** Inhibition of cancer cell proliferation by fruit extracts from four groups of peaches, landraces (L), peaches bred in China (C), peaches bred in Japan and South Korea (JSK) and western improved varieties (WI). Four cancer cell lines, HepG2 (liver cancer), BGC-823 (gastric cancer), A549 (lung cancer), and SW480 (colon cancer), were used. **f** Correlation between inhibition rates of SW480 cell lines by fruit extracts of different peach accessions and the contents of citric acid in these accessions. **g** Inhibition of SW480 cell lines by citric acid, malic acid, and citrate sodium with a concentration of 2000 μg/ml, after treated for 24, 48, and 72 h. **h** Regional Manhattan plots of mGWAS for methylcitric acid (qk109140-2), 2-thiophenecarboxylic acid (qk004847-2), citric acid (mws0281-3), histidinol (qk017487-2), nicotinic acid (mr799), and D-erythro-dihydrosphingosine (mr211) and their overlapping with sweeps related to differentiation, improvement, and domestication at the top of chromosome 5

Since the association signal of malic acid was not ideal, we focused on the mGWAS results of citric acid. Lead SNPs for the citric acid content were located at chromosome 5: 661,951 bp and 661,855 bp in 2015 and 2016, respectively (Fig. 5a), which were different from the *PpALMT1* locus on chromosome 6 that was recently reported to be related to the differentiation between eastern and western peaches [43]. The association interval (Chr. 5: 636,855-686,951 bp) contained three genes, of which two had no functional annotations, and one, *Prupe.5G005700*, encoded a NAD(P)-binding Rossmann-fold superfamily protein with the dehydrogenase/reductase activity. Another gene within the neighboring association region, *Prupe.5G006500*, encoding a V-type proton ATPase subunit F, should also be considered because its homologous genes in apple and orange have been reported to regulate citric acid content [44, 45]. Expression profile of *Prupe.5G006500* in an acid variety and a non-acid variety had a higher correlation with the citric acid content [46] than that of *Prupe.5G005700* (Fig. 5b). We then constructed overexpression vectors for *Prupe.5G005700* and *Prupe.5G006500* and transiently transformed them into tobacco plants. The results showed that *Prupe.5G006500* had a stronger regulatory effect on citric acid content than *Prupe.5G005700* (Additional file 1: Fig. S21). Transient expression in peach flesh also indicated that *Prupe.5G006500* had a positive effect on malic acid and quinic acid contents (Fig. 5c). A 6-bp deletion located in the intron of *Prupe.5G006500* that was strongly associated with the citric acid content in the nature population (*p* < 0.01, Fig. 5d) could be adopted in marker-assisted breeding programs. A SNP in the promoter region of *Prupe.5G006500* may explain the differences in gene expression (Additional file 1: Fig. S22) and citric acid content (Additional file 1: Fig. S23) in peach fruits.

In addition to citric acid, 16 other substances also showed significant differences between eastern and western improved varieties in 2015 and 2016 (Additional file 8: Table S7). Some of them have been reported to have the anti-cancer activity [47–51]. We therefore selected the crude extracts of 176 samples in 2016 for anti-cancer activity evaluation. The results confirmed that inhibition rates of four cancer cells by fruit extracts showed no difference among L, varieties bred in China (C) and Japan and South Korea (JSK), but were significantly higher in the WI group (Fig. 5e). To identify specific anti-cancer substances in the crude extracts, we performed correlation analysis between the cancer cell inhibition rates and the metabolite contents in the 176 accessions. Among the top 100 metabolites with high inhibition activities of the four cancer cells, 49 were constantly detected, including eight annotated metabolites (Additional file 1: Fig. S24; Additional file 32: Table S31). Histidinol showed the highest anti-cancer activity, followed by citric acid, 2-thiophenecarboxylic acid, nicotinic acid, nicotinic acid, and so on. The anti-cancer effect of histidinol has been previously reported [49, 50] and it can also be used as an auxiliary drug to reduce side effects of some anti-cancer drugs [52–54]. The anti-cancer effect of citric acid has also been reported [47, 51] and confirmed by our experiment (Fig. 5f, g). We found that all eight anti-cancer substances showed higher contents in WI than in EI, indicating that varieties from Europe and America could provide higher resistance to these cancer cells. Interestingly, we found that lead SNPs of six substances were located close to each other in a small region on chromosome 5, which overlapped with a differentiation sweep between EI and WI (Fig. 5h). Considering acid content is a major component determining fruit flavor, we speculated that the differentiation of the genome in this associated interval could cause the difference in flavor between the eastern and western improved peach varieties, which could indirectly result in the difference in their anti-cancer activities.

## Discussion

Peach is a delicious and healthy summer fruit in most temperate regions of the world [55]. However, very few reports about its nutritional components and related regulatory genes are available. The emergence of omics technologies, including genome, transcriptome, and metabolome, makes it possible to identify genes related to metabolites in a broader and deeper scope [56, 57]. In this study, we identified 8685 and 10,412 lead SNPs for 582 and 628 metabolites in two seasons, 2015 and 2016, respectively, 12,691 expression-metabolite correlations between 567 metabolites and 1826 expressed genes, and 1792 genes regulated by 9255 cis-eQTLs and 1257 regulated by 285,421 trans-eQTLs (Fig. 1a). Using multi-omics technology, we discovered a regulatory network involved in flavonoid metabolism including eQTLs and hub genes (Fig. 2c). In addition, these rich mGWAS datasets provide useful tools for identifying key genes related to essential metabolites, such as L-valine, quercetin 3-*O*-glucoside, and citric acid (Figs. 3, 4, and 5). However, we should also understand that although the multi-omics method provides a lot of data, most of them are currently hard to utilize. For example, of the 1858 distinct metabolites identified, only 257 (13.8%) could be annotated. Therefore, mGWAS results of the unannotated substances are difficult to be analyzed more deeply for candidate gene identification. Therefore, characterizing these substances through biochemical approaches and then identifying the underlying candidate genes will be possible to construct novel key biological pathways of peach in the future.

Peach originated in China with a wide territory. After a long period of local adaptation, peaches have formed different ecological types with obvious difference in geographical environments. It is well known that plants can adjust the contents of metabolites in response to climate change. For example, Sun et al. [58] found that maize could adapt to temperature variations through the interrelation of plastic responses in the metabolomes and functional traits, such as biomass allocation and carbon and nitrogen status. Kim et al. [59] reported an essential drought-responsive network in which plants trigger a dynamic metabolic flux conversion from glycolysis into acetate synthesis to stimulate the jasmonate (JA) signaling pathway conferring drought tolerance. Therefore, peach landraces represent good materials for studying the roles of metabolites in peach adaptation to different environments. In this study, by analyzing metabolite profiles of different peach landraces, we identified a number of metabolites responsive to low temperature and/or drought stresses. A comparative analysis showed that 25 known metabolites were involved in responses to both low temperature and drought (Additional file 31: Table S30), including betaine and L-valine that have reported roles in stress responses, indicating peach fruits share some common metabolic adaptation to different stress responses. However, different from low temperature response, some primary metabolites, such as malic acid, citric acid, glucose, and sorbitol, were found to be related to drought response. More importantly, some metabolites with no functional annotation but responding strongly to temperature or drought should be focused in future functional and biochemical characterizations to develop corresponding products to regulate the adaptation of plants to different stresses.

In recent years, consumers often complain that most fruits lack rich flavor. It has resulted from the pursuit of increases in yield and storage properties at the expense of flavor and aroma in breeding programs [60]. Based on the analysis of the metabolite contents in different groups of peaches, we revealed the effects of human selection on metabolite classes and their contents in peach fruits. Firstly, we found that contrary to the increase in sugar content, there is a significant decrease in acid content (mainly including malic acid and critic acid), which may result in a more monotonous taste of peach fruit. Secondly, the contents of volatile substances may have indeed decreased, as reported in our previous study [61]. In this study, we found a hotspot of unknown metabolites (Fig. 2a) which was located at the top of chromosome 4 (around 1.4 Mb). After comparing the genetic locations, we found that this genome region contained key QTLs for terpenoid volatiles in our previous research [61]. Therefore, we speculate that these unknown metabolites could be precursors of the terpenoid volatile substances. Analyzing these unknown metabolites, such as pe35270-2, pe37200-1, pe60924-1 (Additional file 6: Table S5), showed that their contents were significantly reduced in improved varieties than landraces. In addition, affected by the breeding object to select low-bitter and low acid fruit [62], the decrease of flavonoid contents in improved varieties also leads to other adverse effects on human health, such as the reduction in antioxidant and anti-cancer activities found in this study. Our study showed that it is feasible to reintroduce the lost nutritional metabolites into elite varieties with the help of metabolomics techniques and carry out more precise molecular design breeding or use genome editing technologies for improvement in future.

Regarding the influence of human selection on the levels of metabolites in peach, in addition to the association signals on chromosome 5 associated with the differentiation between eastern and western peaches analyzed in this study, co-selection among different targets was universal in other regions. This phenomenon has been termed direct and indirect selection of metabolites in tomato [63]. We took the other three obvious hotspots in Fig. 2a as examples and found that the hotspot on chromosome 2 (Chr. 2: 25 Mb) overlapped with the differentiation interval, the hotspot on chromosome 4 (Chr. 4: 11–13 Mb) was under domestication, and the hotspot on chromosome 8 (Chr. 8: 3–5 Mb) was located in the domestication and differentiation interval. In previous studies, QTLs for malic acid were found to be located in the first and third hotspots [64], and the second locus contains a key gene involved in fruit maturity [65]. Thus, in addition to the direct human selection of metabolites for some important breeding objects, a large number of other metabolites are subject to indirect selection.

## Conclusions

In summary, our results represent a comprehensive metabolomic analysis of fruit crops and have improved our understanding of the metabolic response to environmental change and human selection. Our findings emphasize that plant metabolites are crucial not only for plant growth but also for human health. In combination with the genome and transcriptome information, valuable genes and genomic variations associated with metabolites have been identified. The hotspots of leading SNPs associated with beneficial and adverse metabolites for human health have led us to envisage that more precise and targeted breeding technologies should be used in future improvements.

## Methods

### Plant material, growth condition, and sequencing

A diverse worldwide collection of 252 peach accessions, including five wild peaches, four ornamental lines, 77 landraces, and 166 improved varieties maintained at the National Fruit Tree Germplasm Repository (Zhengzhou Fruit Research Institute, Chinese Academy of Agricultural Sciences, China) were used in this study. All fruits were collected in two seasons, 2015 and 2016. During the growing period, all peach cultivars were equally managed. Ripening fruits were randomly collected and pooled for metabolic profiling. Considering the huge alternation/transition occurred in the fruits in 2 weeks before fully maturation, immature fruits (15 days before ripening) from 185 accessions were used for transcriptome sequencing. To determine the optimal maturity period of each variety, we first estimated their maturity period based on previous evaluation results. Fruits were then picked in 20 days before the estimated date with a 5-day interval. All the fruits were stored under −80 °C. Meanwhile, the firmness of fruits was measured to draw a change pattern at different sampling stages. The date with the highest firmness was defined as the suitable ripening time of the varieties in this year. Finally, samples which were picked in 15 days before maturity were selected for transcriptome sequencing. For metabolic responses of peach fruits under drought and cold stresses, the peach variety “Zhong Nong Jin Hui” of 3 years old was used for the stress treatment at 60 days after blooming. After treatment, fruits were sampled and immediately frozen in liquid nitrogen and stored at −70 °C until vacuum freeze-drying. At least five fruits from each treatment were pooled together into one sample. Two independent biological replicates were metabolically profiled and used for transcriptome analysis. To investigate expression patterns of related genes and the metabolite contents related to stress during peach fruit development, fruit samples were taken at five stages (45, 60, 80, 100, and 120 days after full blooming) from peach varieties “Zheng Bai 5-6”. Three biological replicates were collected for each stage. In addition, to identify genes related to acid contents, fruit samples were taken at four stages (30, 60, 75, and 90 days after full blooming) from peach varieties “Zhong You Tao 4#” (non-acid) and “NJC83” (acid).

The peach variety “Dong Xue Mi Tao” and *Nicotiana benthamiana* were used for the transient expression of target genes. Plants were grown in a growth chamber under normal conditions: 22 °C, 16 h light and 8 h dark, 60% relative humidity.

### Metabolite profiling

A previously described relative quantification method of widely targeted metabolites was used to analyze samples [16]. The freeze-dried peach flesh was crushed using a mixer mill (MM 400, Retsch) with zirconia beads for 1 min at 30 Hz. Sixty to eighty milligrams of powder was extracted overnight at 4 °C with 1 ml of 70% aqueous methanol. Following centrifugation at 12,000 rpm for 10 min at 4 °C, the extracts were absorbed (CNWBOND Carbon-GCB SPE Cartridge, 250 mg, 3 ml; ANPEL, Shanghai, China, www.anpel.com.cn) and filtrated (SCAA-104, 0.22 μm pore size; ANPEL, Shanghai, China, www.anpel.com.cn), and then analyzed using an LC-ESI-MS/MS system (HPLC, Shim-pack UFLC Shimadzu CBM30A system, www.shimadzu.com.cn; MS, Applied Biosystems 4500 QTRAP, www.appliedbiosystems.com.cn/). The analytical conditions were as follows: HPLC column, Waters ACQUITY UPLC HSS T3 C18 (1.8 μm, 2.1 mm × 100 mm); solvent system, water (0.04% acetic acid): acetonitrile (0.04% acetic acid); gradient program, 95:5 V/V at 0 min, 5:95 v/v at 11.0 min, 5:95 v/v at 12.0 min, 95:5 v/v at 12.1 min, 95:5 v/v at 15.0 min; flow rate, 0.35 ml/min; temperature, 40 °C; injection volume, 2 μl. The effluent was alternatively connected to an ESI-triple quadrupole-linear ion trap (QTRAP) MS.

Linear ion trap (LIT) and triple quadrupole (QQQ) scans were acquired on a triple quadrupole-linear ion trap MS (QTRAP) using an API 4500 QTRAP LC/MS/MS System, which was equipped with an ESI Turbo Ionspray interface operated in positive ion mode and controlled by Analyst 1.6.2 software (ABSciex). The ESI source operation parameters were as follows: ion source, turbo spray; source temperature, 550 °C; negative ion spray voltage (IS), 4500 V; ion source gas I (GSI), gas II (GSII), and curtain gas (CUR) were set at 55, 60, and 25 (35) psi, respectively; and the collision gas (CAD) was high (medium). Instrument tuning and mass calibration were performed with 10 and 100 mmol/l polypropylene glycol solutions in QQQ and LIT modes. The QQQ scans were acquired as multiple reaction monitoring (MRM) experiments with the collision gas (nitrogen) set to 5 psi. The declustering potential (DP) and collision energy (CE) for individual MRM transitions were performed with further DP and CE optimization. A specific set of MRM transitions was monitored for each period according to the metabolites that were eluted within this period.

Using the above method, a total of 70 representative samples (35 in 2015 and 35 in 2016 years) were selected and carried out metabolome library construction. The result showed that 2151 substances could be identified. After performing quality control, a total of 1858 metabolites were found to be stable. These 1858 metabolites were used as the references to identify metabolites in the 252 samples over 2 years.

### Browning degree

Samples collected in 2015 were ground thoroughly and treated with 90 °C for 60 s. Then 0.1% pectase was added to the mixture for incubation for 40 min under 50 °C. The mixture was then centrifuged at 5000×*g* for 10 min, and 5 mL supernatant was taken and diluted to 10 ml with 95% ethanol. The solution was centrifuged at 7800×*g* for 10 min. The absorbance of the supernatant was measured with a spectrophotometer at 420 nm (A0). The supernatant was then heated at 80 °C for 8 h. The absorbance was measured again when the supernatant became cool (A1). The browning degree was evaluated as the difference between the two values (A1-A0).

### Measurement of inhibition activities on four cancer cell proliferation

A total of 176 out of 252 peach accessions were selected to analyze the inhibition activities of fruit extracts on four cancer cell lines. For each accession, a total of 10 mature fruits were picked, and the mesocarp of the fruit was frozen and ground into powder in liquid nitrogen. The flesh powder was then extracted with deionized water and methanol with a ratio of 1:3 (m/v). During the extraction, ultrasound was used to increase the extraction effects for 12 min. The extracts were centrifuged at 4000 rpm for 10 min at 4 °C to collect supernatants. The methanol extracts were treated by rotary evaporation at 45 °C until no methanol was retained. The obtained solution was stored at −20 °C for cell line experiments. The concentration of the solution was calculated at about 320 g·L^−1^.

Human liver cancer cells (HepG2), gastric cancer cells (BGC823), lung cancer cells (A549), and colon cancer cells (SW480) were purchased from the Chinese Academy of Sciences, Shanghai, China. HepG2 was cultivated in the RPMI-1640 medium (Thermo Fisher Scientific, Waltham, MA), and BGC823, A549, and SW480 were cultivated in the DMEM medium (Thermo Fisher Scientific, Waltham, MA). All cells were supplemented with 10% (v/v) fetal bovine serum and 1% penicillin-streptomycin antibiotic mix (Beyotime, Shanghai, China) and maintained at 37 °C, 100% humidity, and 5% CO_2_ incubator over the entire evaluation period.

The anti-cancer activities of different peach extracts were compared by testing their capacities to inhibit the proliferation of four cell lines using the CKK-8 assay. After 100 μL of each cell culture was placed in a 96-well plate at a concentration of 1 × 10^5^ cells/mL, cells were cultivated in a 5% CO_2_ incubator at 37 °C for 4 h. Extracted samples of peach fruit were added to the cell cultures. Each culture was incubated for 48 h in a 5% CO_2_ incubator at 37 °C, and 10 μL of CKK-8 (Beyotime, Shanghai, China) was then added to the wells. After 3 h of incubation, cell proliferation was determined by its absorbance at 450 nm. Control cultures received the extraction solution minus the fruit extracts, and blank wells contained 100 μL of growth medium with no cells. At least three replications for each sample were used to determine cell proliferation. Finally, cell proliferation inhibition rate was expressed using the following formula: % inhibition rate = [(Mean absorbance of control − Mean absorbance of treated cells)/(Mean absorbance of control − Mean absorbance of blank)] × 100%. In addition, citric acid, malic acid, and citrate sodium were also used with different concentrations to evaluate their inhibition activity.

### Environmental classification

To understand the influence of the environment on metabolite profiles in peach fruit, rainfall, and temperature records in the origins of 76 landrace varieties were obtained from China Meteorological Data Service Center (http://data.cma.cn/). In a few cases, the nearest meteorological station data were considered if the meteorological data of the original place is missing. The average values of rainfall and temperature in different origins in the peach growing season (from April to October) from 1961 to 2010 (50 years) were used for subsequent classification. The rainfall level in the specific region was classified into five categories: Level 1, average annual rainfall between 1 and 370 mm; Level 2, average annual rainfall between 370 and 740 mm; Level 3, average annual rainfall between 740 and 1110 mm; Level 4, average annual rainfall between 1110 and 1,480 mm; Level 5, average annual rainfall between 1480 and 1850 mm. Similarly, five categories were used to evaluate the temperature in the specific region: Level 1, the annual average temperature between 7 and 10 °C; Level 2, the annual average temperature between 10 and 13 °C; Level 3, the annual average temperature between 13 and 16 °C; Level 4, the annual average temperature between 16 and 19 °C; Level 5, the annual average temperature between 19 and 23 °C (Additional file 2: Table S1).

### SNP identification

Genome resequencing was performed for the 252 peach accessions used in this study. Total genomic DNA was extracted from young leaves using the CTAB method [66]. Library construction and sequencing were same as in the previous report [21]. Raw data were cleaned and aligned to the peach reference genome v2.0 [67] for SNP calling using the GATK software [68]. The called 2,685,327 SNPs were filtered by removing those with MAF < 0.05, missing rate > 0.2, and Hardy-Weinberg equilibrium (HWE) *p*-value < 1.0 × 10^−6^. Finally, a total of 486,009 SNPs were retained.

### Metabolome data analysis

Principal component analysis (PCA) was performed with metabolite data of 252 peach accessions. Identification of differential accumulation of metabolites between different varieties was determined by partial least squares discriminate analysis (PLS-DA) with VIP values (variable importance for the projection) ≥ 1. PCA and PLS-DA were performed with SIMCA-P version 14.0.

### RNA-Seq data analysis

Total RNA of peach fruit flesh was extracted by an RNA Extraction Kit (Aidlab, Beijing, China). First- and second-strand complementary DNA (cDNA) was synthesized using a cDNA Synthesis System kit (Takara, Dalian, China), following the manufacturer’s protocol. The resulting double-strand cDNA was purified, and adapters were ligated to the short fragments. The constructed RNA-Seq libraries were sequenced on the Illumina HiSeq 2500 platform in paired-end 150-bp mode. Low-quality reads were filtered from the raw reads, and an average of 46.78 million cleaned reads were obtained for each library. Cleaned reads were mapped to the peach reference genome (Version 2.1) using TopHat v2.1.0 [69] with default parameters, and Cufflinks v2.1.1 [70] was used to quantify expression (FPKM) values for each gene among samples.

### Genome-wide association study

A total of 486,009 SNPs were used for the genome-wide association study. Population structure was modeled by admixture (Version 1.2.3) [71], and TASSEL (version 3.0) [72] was used to calculate the kinship value. mGWAS was performed using the LMM (linear mixed model) implemented in TASSEL. The genome-wide significance thresholds was determined using the Bonferroni test threshold (*p* = 3.19 × 10^−7^), and the lead SNP within the 100-kb window for each metabolite was extracted as one signal.

The hotspots of mGWAS were detected according to previous study [73]. Firstly, we investigated the distribution of lead SNPs of different metabolites in 1-Mb windows along the genome. Then, a permutation test was used to calculate the threshold of hotspot identification. The results of 1000 permutations showed that, with *p* < 0.05, the cutoff number of significant lead SNPs per Mb would be 50 in 2015 and 59 in 2016.

### eQTL analysis

A total of 13,050 genes expressed in at least 80% of the accessions (FPKM ≥ 1) were selected to perform eQTL analysis with the 486,009 SNPs. The association between SNPs and the gene expression was calculated using the Matrix eQTL software [74] at a rigorous Bonferroni-corrected *α* = 0.05 for identification of trans-eQTLs (*P* = 1.99 × 10^−6^) and cis-eQTLs (*P* = 1.99 × 10^−3^). The identified eQTLs were categorized into cis-eQTLs (located within 30 kb from the transcription start site of the target genes) and trans-eQTLs. We also identified the hotspots of eQTLs using permutation test and Bonferroni correction. The window size and the *P* values were set to 1 Mb and 0.05, respectively.

### Correlation analysis between metabolome and transcriptome profiles

Metabolome and transcriptome profiles from 185 peach accessions were used to identify genes whose expression profiles were significantly correlated with the metabolite contents. Significant correlations between contents of 1858 metabolites and expression levels of 22,374 genes in the population were identified, with a threshold of *p* ≤ 2.23 × 10^−6^ (corresponding to a Bonferroni-corrected *α* level less than 0.05).

### Co-expression modules identification

A gene co-expression network was constructed using the Weighted Correlation Network Analysis (WGCNA) R package (v1.70-3), with a correlation matrix soft-thresholding power *β* of 8 [75]. A total of 22,374 genes were screened using the goodSamplesGenes function, and the resulting 22,030 genes were used for the WGCNA analysis. Finally, a total of 14,476 genes were assigned to 47 modules.

### Detection of domestication, improvement, and differentiation sweeps

To identify genomic regions affected by domestication, improvement, and differentiation, we compared the *F*_*ST*_ in 100-kb windows with a step size of 10 kb using VCFtools (version 0.1.12b) [76]. For domestication, *F*_*ST*_ values of wild and ornamental groups in contrast to landraces were calculated. For improvement, *F*_*ST*_ values were calculated between landraces and improved varieties. For differentiation, *F*_*ST*_ values were calculated between eastern and western improved varieties. The top 5% of windows or regions with the highest *F*_*ST*_ values were defined as selective sweeps.

### Transient expression of peach genes in N. benthamiana and peach

Transient overexpression vectors were constructed by directionally inserting the full-length cDNAs into the entry vector pSAK277 according to Zhou et al. [77]. The constructs were then transformed into *Agrobacterium tumefaciens* (EHA105). Positive clones were selected and grown to optimal density of 1.6 at 600 nm (OD_600nm_) in 50 ml of LB medium (5 g/l yeast extract, 10 g/l tryptone, 10 g/l NaCl), washed with washing buffer (10 mM 2-(N-morpholino) ethanesulfonic acid [MES] [pH 5.6]), and resuspended in MMA buffer (10 mM MES [pH 5.6], 10 mM MgCl_2_, 100 mM acetosyringone) to an OD600 of 0.8. The culture was incubated for 2 h at room temperature, and 1 ml of culture was infiltrated into the underside of 6-week-old *N. benthamiana* leaves or immature peach fruits. The samples were then rinsed three times with sterile water and cultured on MS medium. The metabolite contents and gene expression levels were measured after 3 days of injection. Transient expression treatments were repeated three times with six tobacco leaves and five peach fruits in each replicate. Measurements of valine and citric acid contents were conducted by targeted high-throughput LC-MS/MS approach and the methods described in our previous study [21], respectively. In this study, the transient system was used in tobacco to verify the functions of key genes regulating critic acid content in flesh. Meanwhile, the system was used in peach to verify the functions of gene involved in valine and critic acid biosynthesis.

### Drought and cold treatments

The drought and cold treatment experiments were carried out on peach seedlings of 3 years old. The water content was maintained in optimal conditions for all plants prior to drought stress treatment. During the treatment period, stressed plants had no water supply, whereas control plants were watered every 3 days to field capacity under the greenhouse. Fruit samples used for metabolite analysis were collected at 0, 1, 3, and 6 days of the drought stress. For cold treatment, control plants were grown in a growth chamber under normal conditions (22 °C with 16 h light and 8 h dark, 60% relative humidity), and treated plants were transferred to 4 °C with the abovementioned photoperiod. Fruit samples used for metabolite and RNA-seq analyses were collected at 0, 3, and 6 days after treatment. All collected samples were immediately frozen in liquid nitrogen and then stored at −80 °C till use. Each treatment consisted of two biological replicates and each replicate contained five plants grown in the same conditions.

### Statistical analysis

The coefficient of variation values were calculated for each metabolite in the population as follows: *s*/*m*, where *s* and *m* are the standard deviation and mean of each metabolite in the population, respectively. Broad-sense heritability (*H*^*2*^) was estimated using the following formula: *H*^*2*^= var(G)/var(G)+var(E), where var(G) and var(E) are the variances derived from genetic and environmental effects, respectively.

## Supplementary Information

Additional file 1: Figure S1. Broad-sense heritability (a) and coefficient of variations (b) of all metabolites detected across the two seasons. Figure S2. Correlation of annotated metabolites between seasons 2015 and 2016. (a) Number of annotated metabolites with different correlation coefficients. (b) Categories of annotated metabolites that had a high correlation between the two seasons. Figure S3. Volcano plot to identify differential metabolites between wild and cultivated peaches in 2015 (a) and 2016 (b). Figure S4. Volcano plot to identify differential metabolites between landraces and improved varieties in 2015 (a) and 2016 (b). Figure S5. Volcano plot to identify differential metabolites between eastern and western improved varieties in 2015 (a) and 2016 (b). Figure S6. Distribution of explained variation (R^2^) of associated SNPs in 2015 (a) and 2016 (b). Figure S7. Distribution of associated SNPs across peach chromosomes. Figure S8. Distribution of detected genes in transcriptomes in all peach accessions. Figure S9. Volcano plot to identify differential expressed genes between wild and cultivated peaches (a), landraces and improved varieties (b), eastern and western improved varieties (c) in 2016. Figure S10. Heatmap of differential expressed genes in W, L, EI, and WI groups. Figure S11. Enrichment of KEGG pathways in differentially expressed genes associated with peach domestication (a), improvement (b) and differentiation (c). Figure S12. Number of expressed genes correlated with the content of each metabolite. Figure S13. Co-expression network modules constructed using weighted correlation network analysis (WGCNA) based on gene expression values. Each color indicates a different module. Figure S14. Genome screening of selective sweeps in peach and their overlaps with associated SNPs of flavonoids. (a) Selective sweeps during peach domestication. (b) Selective sweeps during peach improvement. (c) Selective sweeps during differentiation between eastern and western improved varieties. Figure S15. Identification of differential metabolites according to temperature levels in the growing period in the origin places of peaches. (a) Heatmap of relative contents of all annotated metabolites between level 1 and level 4&5 populations of peach. (b) PCA plot of landraces according to the differential metabolites detected between level 1 and level 4&5 populations of peach. (c) Boxplot of L-valine contents in level 1 and level 4&5 populations. (d) Boxplot of UDPG contents in level 1 and level 4&5 populations. Figure S16. Identification of differential metabolites according to rainfall levels in the growing period in the origin places of peaches. (a) Heatmap of relative contents of all annotated metabolites between level 1 and level 4&5 populations of peach. (b) PCA plot of landraces according to the differential metabolites detected between level 1 and level 4&5 populations of peach. (c) Boxplot of L-arginine contents in level 1 and level 4&5 populations. (d) Boxplot of betaine contents in level 1 and level 4&5 populations. Figure S17. Volcano plots to identify annotated metabolites responding to cold. (a) Differential metabolites identified according to different temperatures of peach origins. (b) Differential metabolites identified in fruits of peach varieties ‘Zhong Nong Jin Hui’ treated with low temperature (4 °C). (c) Differential metabolites regulated by both long and short cold inductions. Figure S18. Volcano plots to identify annotated metabolites responding to drought. (a) Differential metabolites identified according to different rainfalls of peach origins. (b) Differential metabolites identified in fruits of peach varieties ‘Zhong Nong Jin Hui’ treated with drought. (c) Differential metabolites regulated by both long and short drought treatments. Figure S19. Proline contents in leaves treated by spraying water (WT) and L-valine solution (50 mg/L) in ‘Shenzhou Li He Shui Mi’ peach trees of 15-leaves old that was induced by -4 °C for 24 or 72 hours. Figure S20. L-valine contents and expression profiles of genes located in the mQTL region of L-valine on chromosome 4 during fruit development. Figure S21. Citric acid contents in lines transiently overexpressing *Prupe.5G005700* or *Prupe.5G006500* in tobacco. Figure S22. Relative expression of *Prupe.5G006500* in peaches with different genotypes at position Chr. 5: 730,270 bp. Figure S23. Variation detection of *Prupe.5G006500* gene associated with citric acid contents in peach fruit. Figure S24. Venn diagram of the top 100 metabolites with high inhibition activities to the four cancer cells.

Additional file 2: Table S1. List of 252 peach accessions used in this study.

Additional file 3: Table S2. Widely targeted metabolite analysis in peach fruits.

Additional file 4: Table S3. Detailed information of metabolites evaluated in 2015 related to Fig. 1d and e.

Additional file 5: Table S4. Significantly changed metabolites in fruits between wild and cultivated peaches (landraces and improved varieties) in 2015 (sheet 1) and 2016 (sheet 2).

Additional file 6: Table S5. Significantly changed metabolites in fruits between landraces and improved varieties in 2015 (sheet 1) and 2016 (sheet 2).

Additional file 7: Table S6. Significantly changed metabolites in fruits between eastern and western improved varieties in 2015 (sheet 1) and 2016 (sheet 2).

Additional file 8: Table S7. Significantly changed metabolites in fruits between eastern and western improved varieties in both 2015 and 2016.

Additional file 9: Table S8. List of lead SNPs detected in peach mGWAS in the two seasons.

Additional file 10: Table S9. Statistics of lead SNPs associated with each metabolite in the two seasons.

Additional file 11: Table S10. Number of associated SNPs in each non-overlapping 1-Mb sliding windows across the peach genome.

Additional file 12: Table S11. List of the 185 accessions used in transcriptome sequencing and eQTL analysis.

Additional file 13: Table S12. Differentially expressed genes detected during domestication (sheet 1), improvement (sheet 2), and differentiation (sheet 3).

Additional file 14: Table S13. Correlation between gene expression and metabolite contents evaluated in 2016.

Additional file 15: Table S14. List of candidate genes within 25-kb flanking regions of the lead SNPs associated with metabolites identified in 2016.

Additional file 16: Table S15. Gene co-expression modules constructed based on gene expression profiles.

Additional file 17: Table S16. KEGG pathways enriched in each of the gene co-expression modules.

Additional file 18: Table S17. Metabolites evaluated in 2016 correlated with different gene co-expression modules.

Additional file 19: Table S18. List of identified cis-eQTLs.

Additional file 20: Table S19. List of identified trans-eQTLs.

Additional file 21: Table S20. List of eQTL hotspots.

Additional file 22: Table S21. Browning indexes (△A) of the 186 peach accessions.

Additional file 23: Table S22. Correlation between metabolite contents and browning indexes.

Additional file 24: Table S23. Selective sweeps related to peach domestication and improvement, and differentiation between eastern and western improved varieties.

Additional file 25: Table S24. Overlap of the lead SNPs of mGWAS in 2016 with selective sweeps.

Additional file 26: Table S25. Correlation between quercetin 3-O-glucoside contents and expression profiles of genes in Chr. 5: 0-5 Mb in peach fruits at different developmental stages.

Additional file 27: Table S26. Significantly changed metabolites in different levels of cold environments.

Additional file 28: Table S27. Significantly changed metabolites in different levels of drought environments.

Additional file 29: Table S28. Metabolite content changes in peach fruits after cold treatment.

Additional file 30: Table S29. Metabolite content changes in peach fruits after drought treatment.

Additional file 31: Table S30. Metabolites responsive to both drought and low temperature.

Additional file 32: Table S31. List of metabolites highly correlated with inhibition rates of all four cancer cell lines.

Additional file 33. Review history.

## Data Availability

Raw Illumina sequencing data from this study have been submitted to NCBI Sequence Read Archive (SRA) under accession PRJNA197462 [78], PRJNA281983 [79], PRJNA388029 [80], PRJNA504509 [81], PRJNA509595 [82], PRJNA630113 [83], and PRJNA680197 [84]. Details about the samples can also be found in Additional file 2: Table S1.
