# Supplementary material for: Combined nature and human selections reshaped peach fruit metabolome
Source: Genome Biol. 2022 Jul 4;23:146. doi: 10.1186/s13059-022-02719-6 (PMC9254577; doi:10.1186/s13059-022-02719-6)
Supplement: Supplementary file 1 — Additional file 1: Figure S1. Broad-sense heritability (a) and coefficient of variations (b) of all metabolites detected across the two seasons. Figure S2. Correlation of annotated metabolites between seasons 2015 and 2016. (a) Number of annotated metabolites with different correlation coefficients. (b) Categories of annotated metabolites that had a high correlation between the two seasons. Figure S3. Volcano plot to identify differential metabolites between wild and cultivated peaches in 2015 (a) and 2016 (b). Figure S4. Volcano plot to identify differential metabolites between landraces and improved varieties in 2015 (a) and 2016 (b). Figure S5. Volcano plot to identify differential metabolites between eastern and western improved varieties in 2015 (a) and 2016 (b). Figure S6. Distribution of explained variation (R2) of associated SNPs in 2015 (a) and 2016 (b). Figure S7. Distribution of associated SNPs across peach chromosomes. Figure S8. Distribution of detected genes in transcriptomes in all peach accessions. Figure S9. Volcano plot to identify differential expressed genes between wild and cultivated peaches (a), landraces and improved varieties (b), eastern and western improved varieties (c) in 2016. Figure S10. Heatmap of differential expressed genes in W, L, EI, and WI groups. Figure S11. Enrichment of KEGG pathways in differentially expressed genes associated with peach domestication (a), improvement (b) and differentiation (c). Figure S12. Number of expressed genes correlated with the content of each metabolite. Figure S13. Co-expression network modules constructed using weighted correlation network analysis (WGCNA) based on gene expression values. Each color indicates a different module. Figure S14. Genome screening of selective sweeps in peach and their overlaps with associated SNPs of flavonoids. (a) Selective sweeps during peach domestication. (b) Selective sweeps during peach improvement. (c) Selective sweeps during differentiation between eastern and [file 13059_2022_2719_MOESM1_ESM.docx]

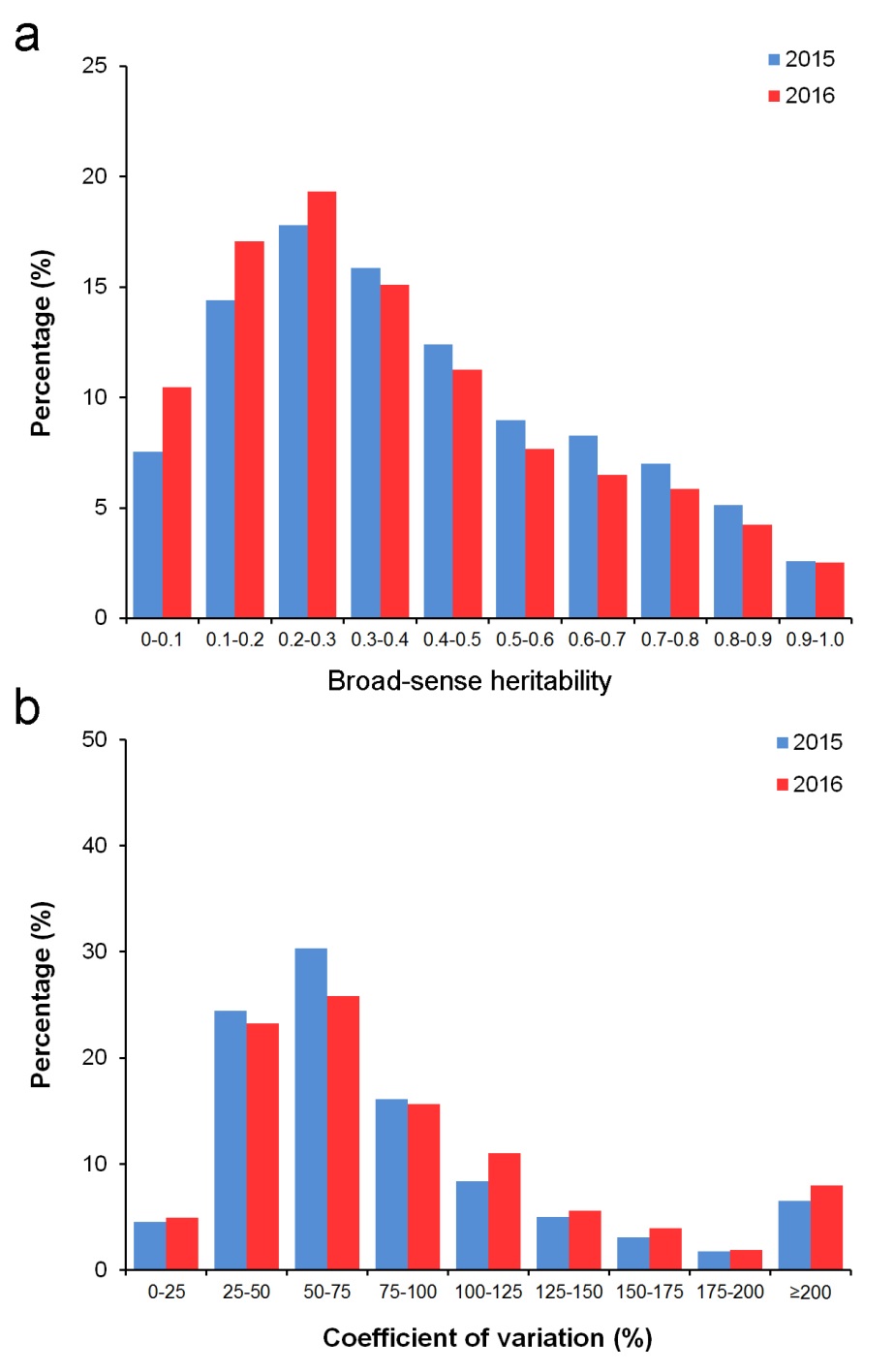


Fig. S1. Broad-sense heritability (a) and coefficient of variations (b) of all metabolites detected across the two seasons.


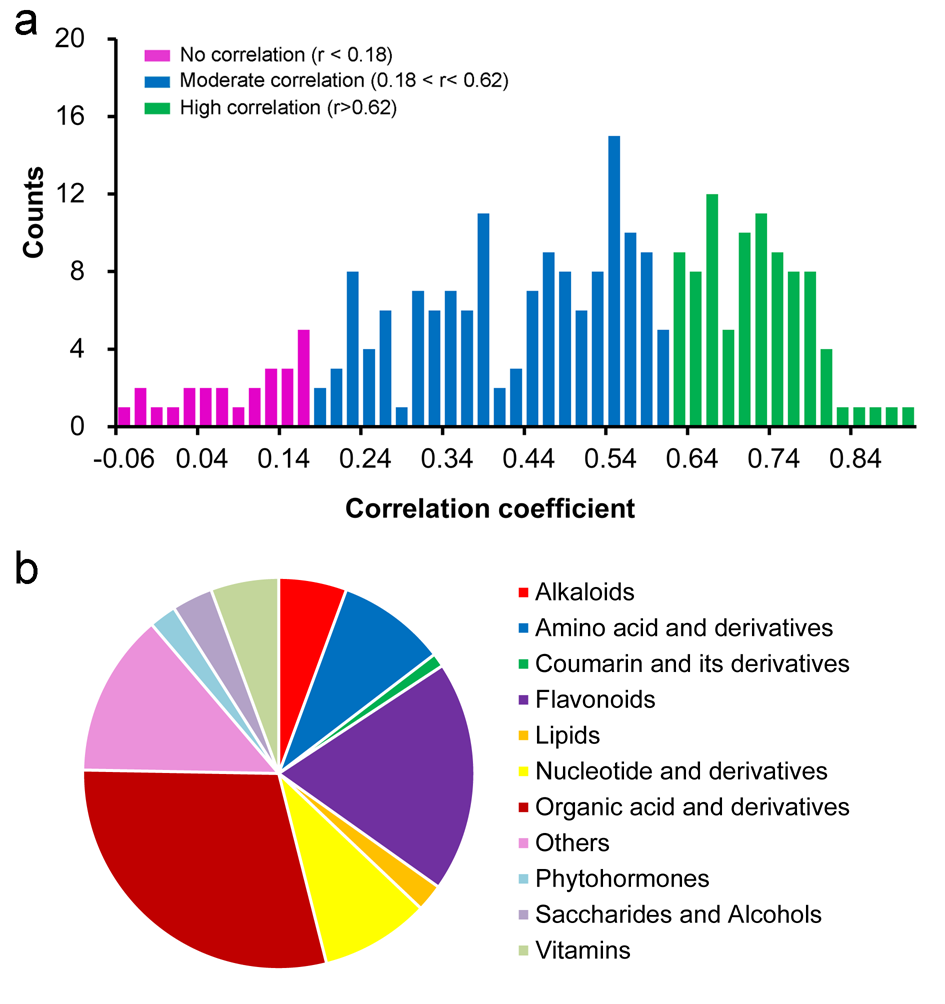


Fig. S2. Correlation of annotated metabolites between seasons 2015 and 2016. (a) Number of annotated metabolites with different correlation coefficients. (b) Categories of annotated metabolites that had a high correlation between the two seasons.


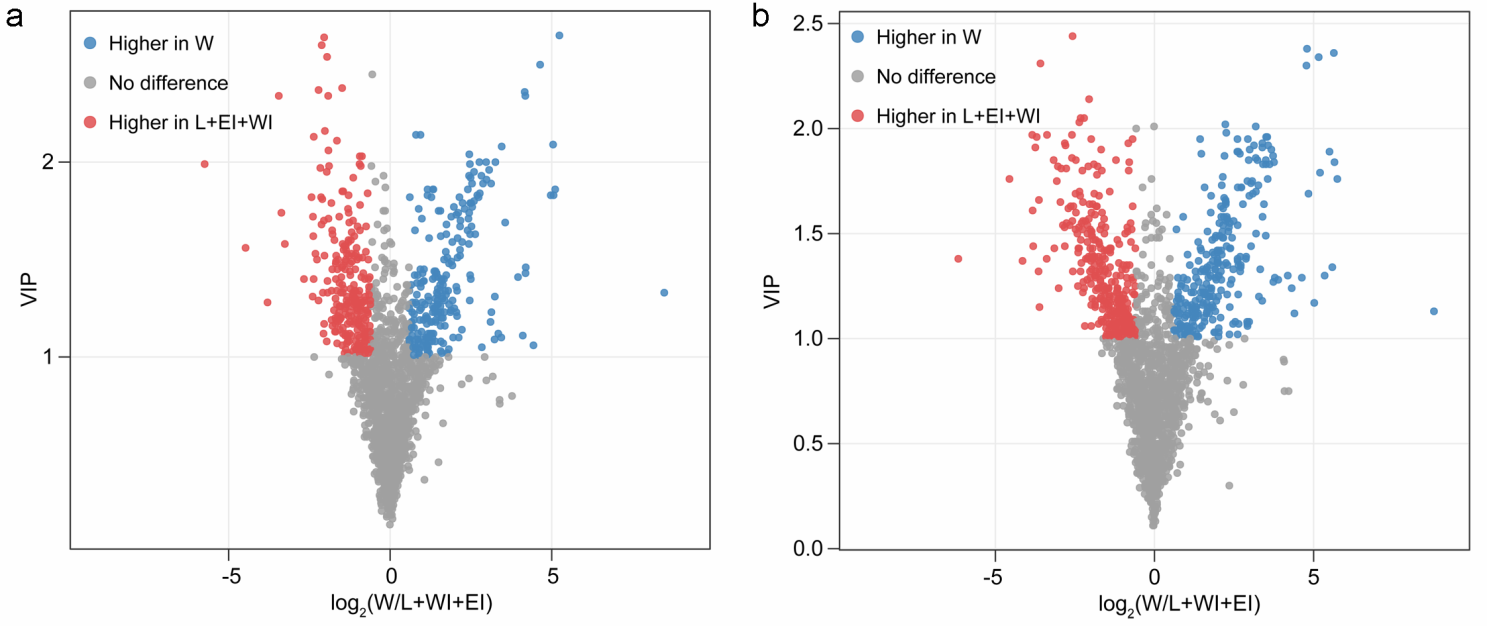


Fig. S3. Volcano plot to identify differential metabolites between wild and cultivated peaches in 2015 (a) and 2016 (b).


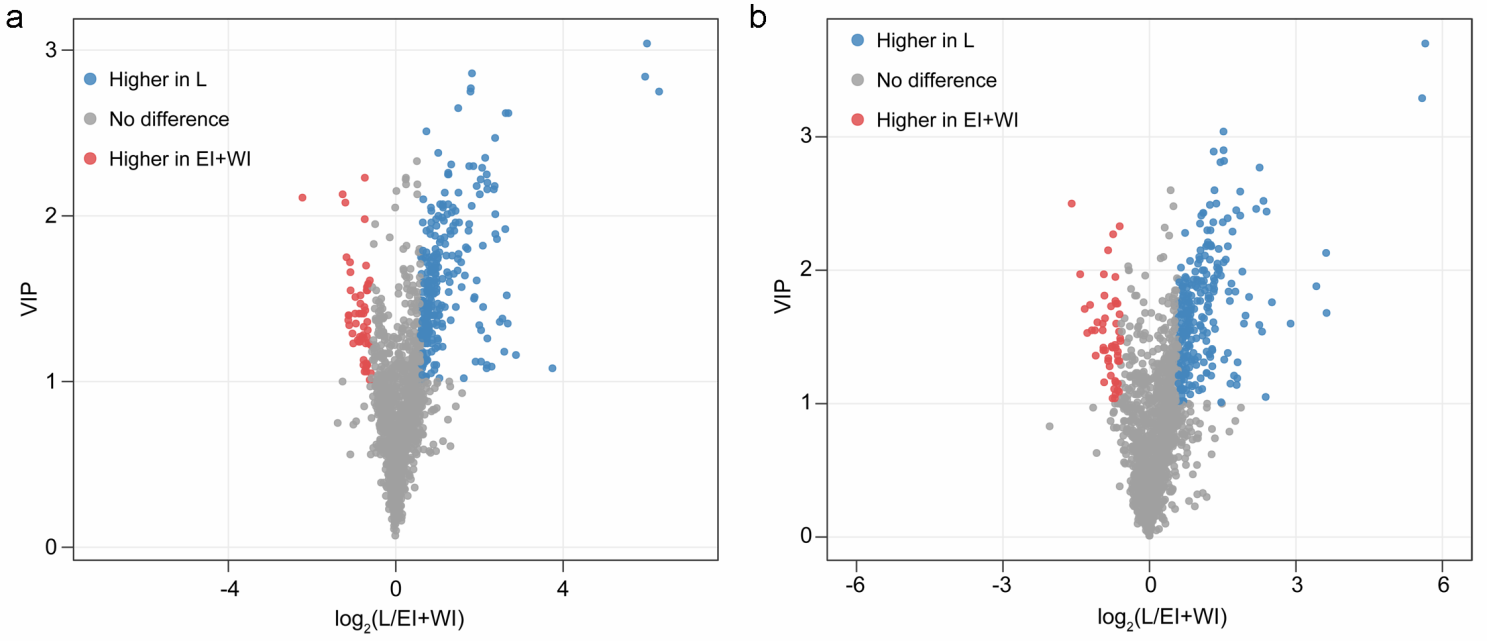
 Fig. S4. Volcano plot to identify differential metabolites between landraces and improved varieties in 2015 (a) and 2016 (b).


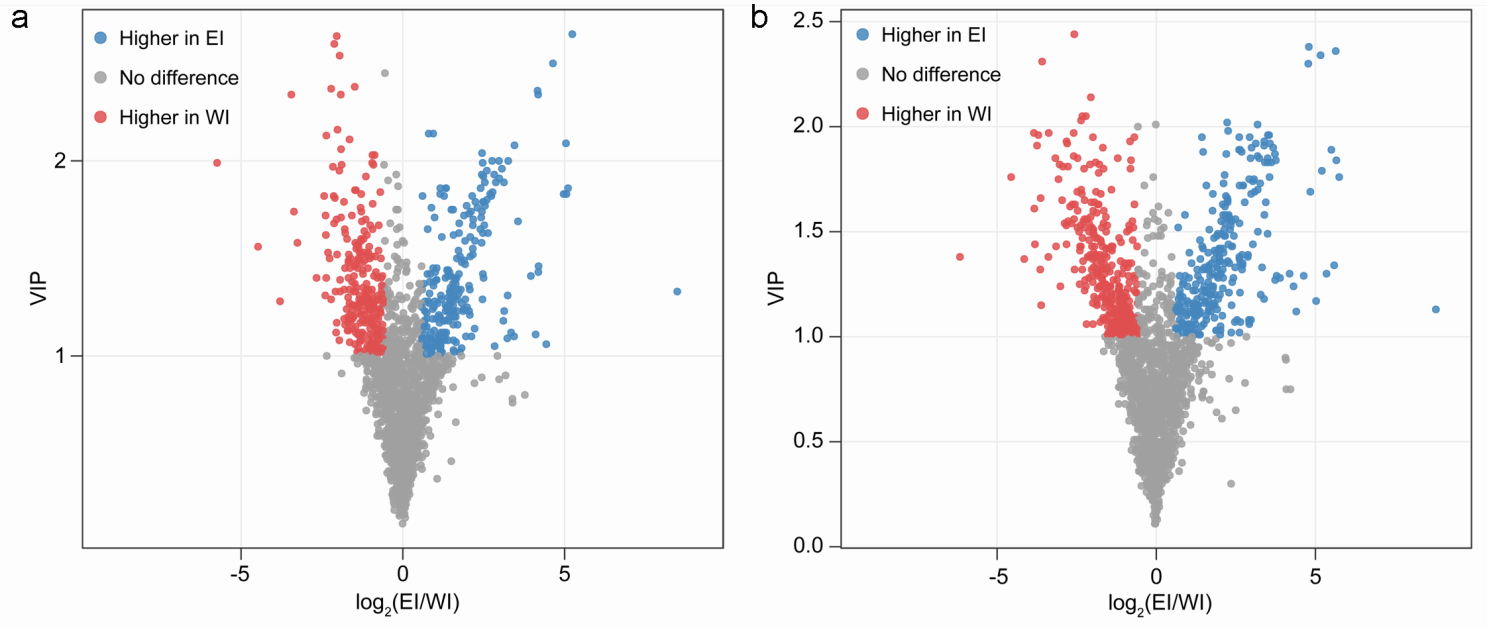


Fig. S5. Volcano plot to identify differential metabolites between eastern and western improved varieties in 2015 (a) and 2016 (b).


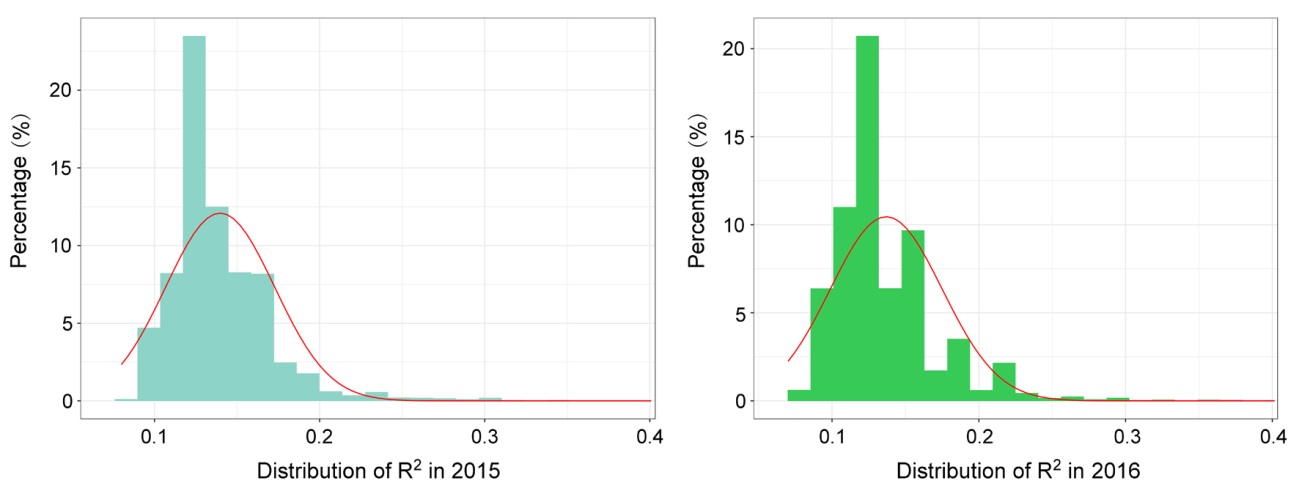


Fig. S6. Distribution of explained variation (R^2^) of associated SNPs in 2015 (a) and 2016 (b).


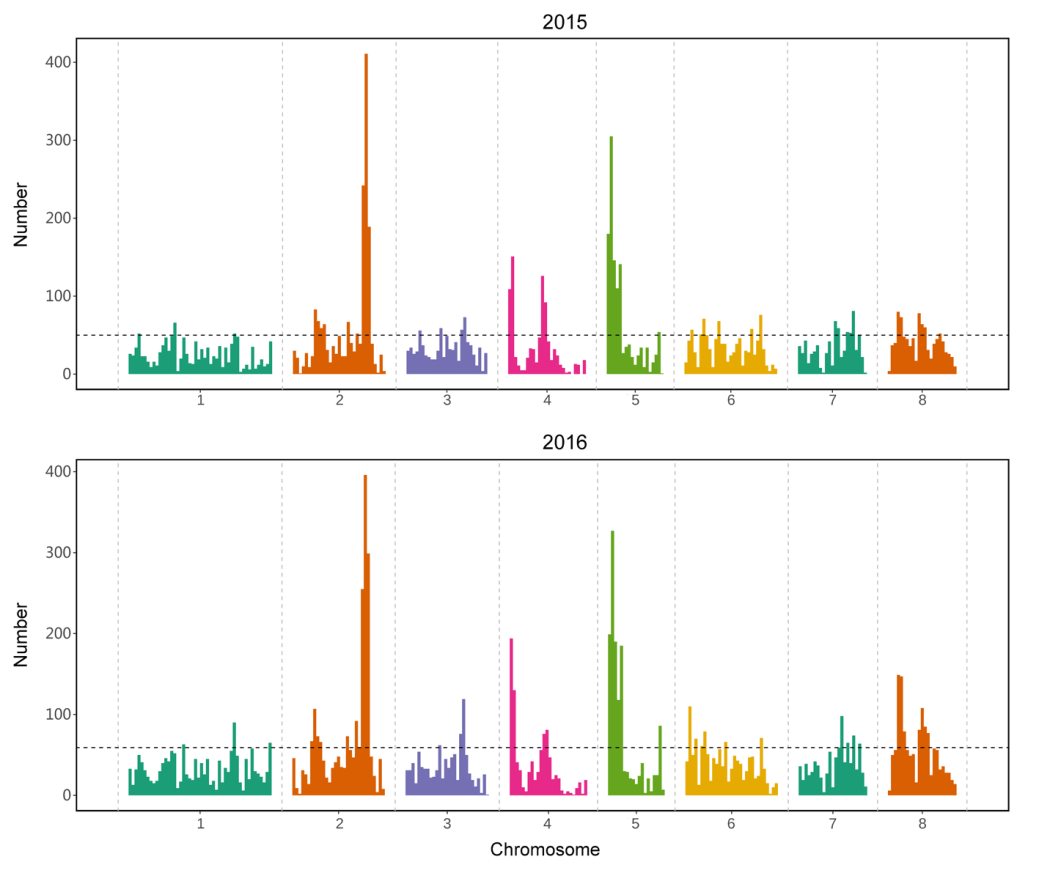


Fig. S7. Distribution of associated SNPs across peach chromosomes.


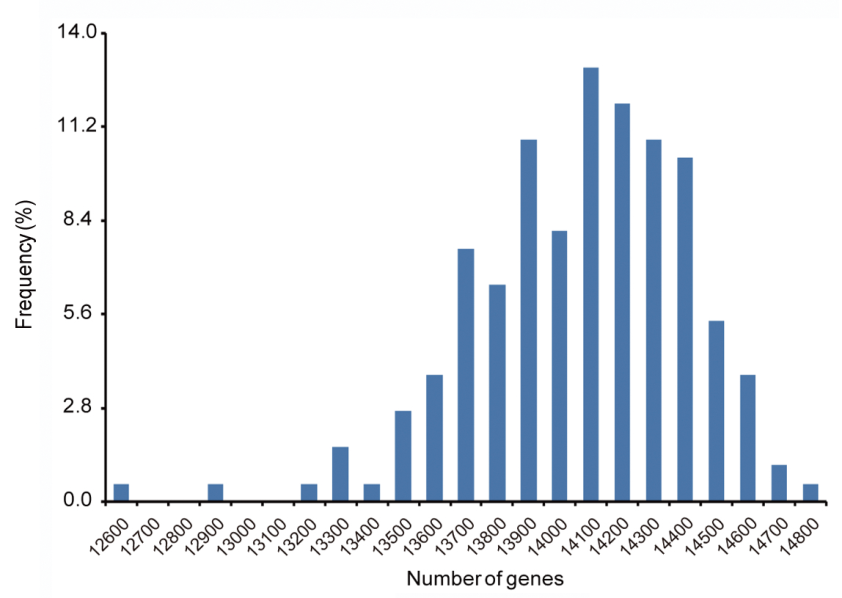


Fig. S8. Distribution of detected genes in transcriptomes in all peach accessions.


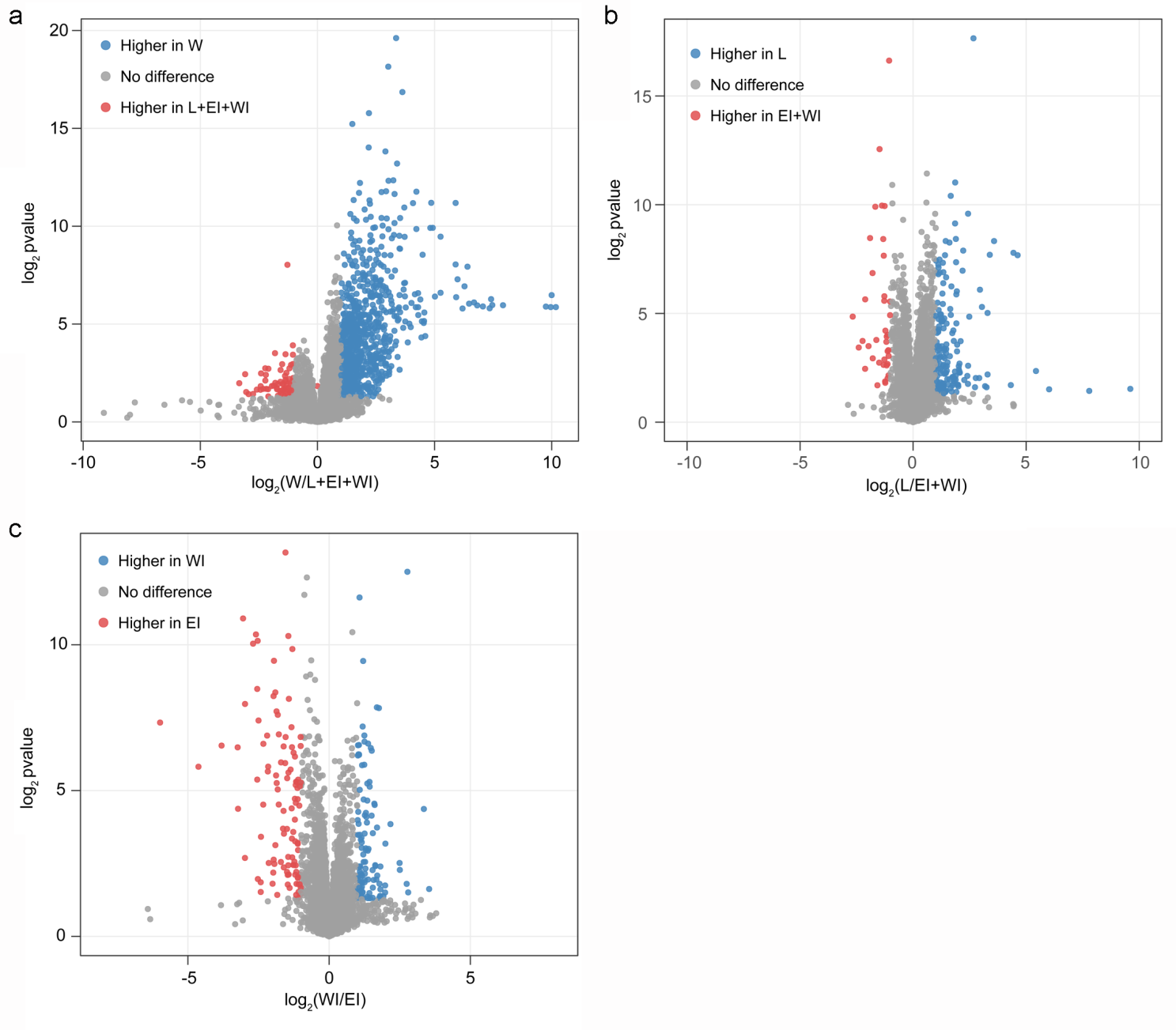


Fig. S9. Volcano plot to identify differential expressed genes between wild and cultivated peaches (a), landraces and improved varieties (b), eastern and western improved varieties (c) in 2016.


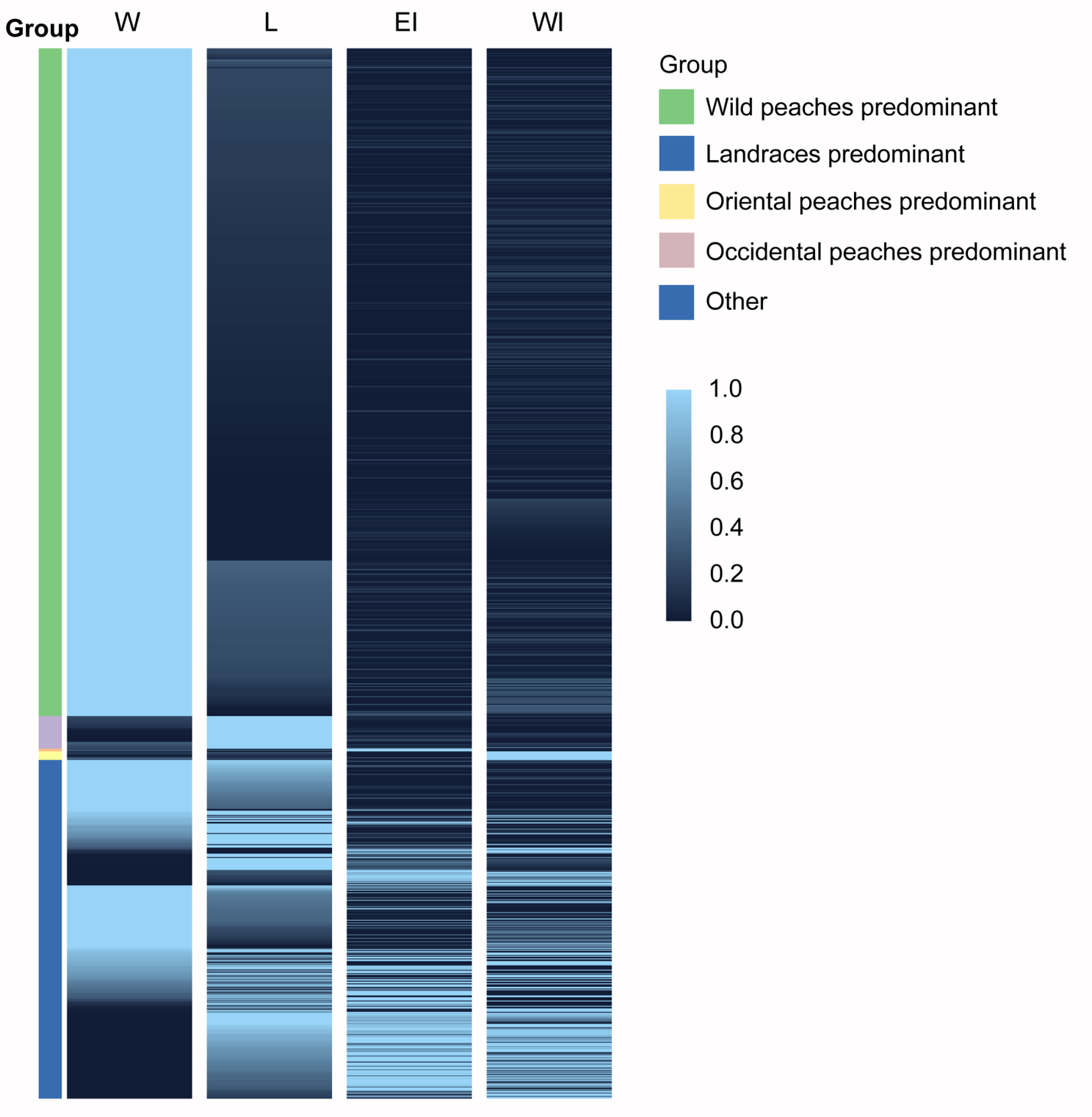


Fig. S10. Heatmap of differential expressed genes in W, L, EI, and WI groups.


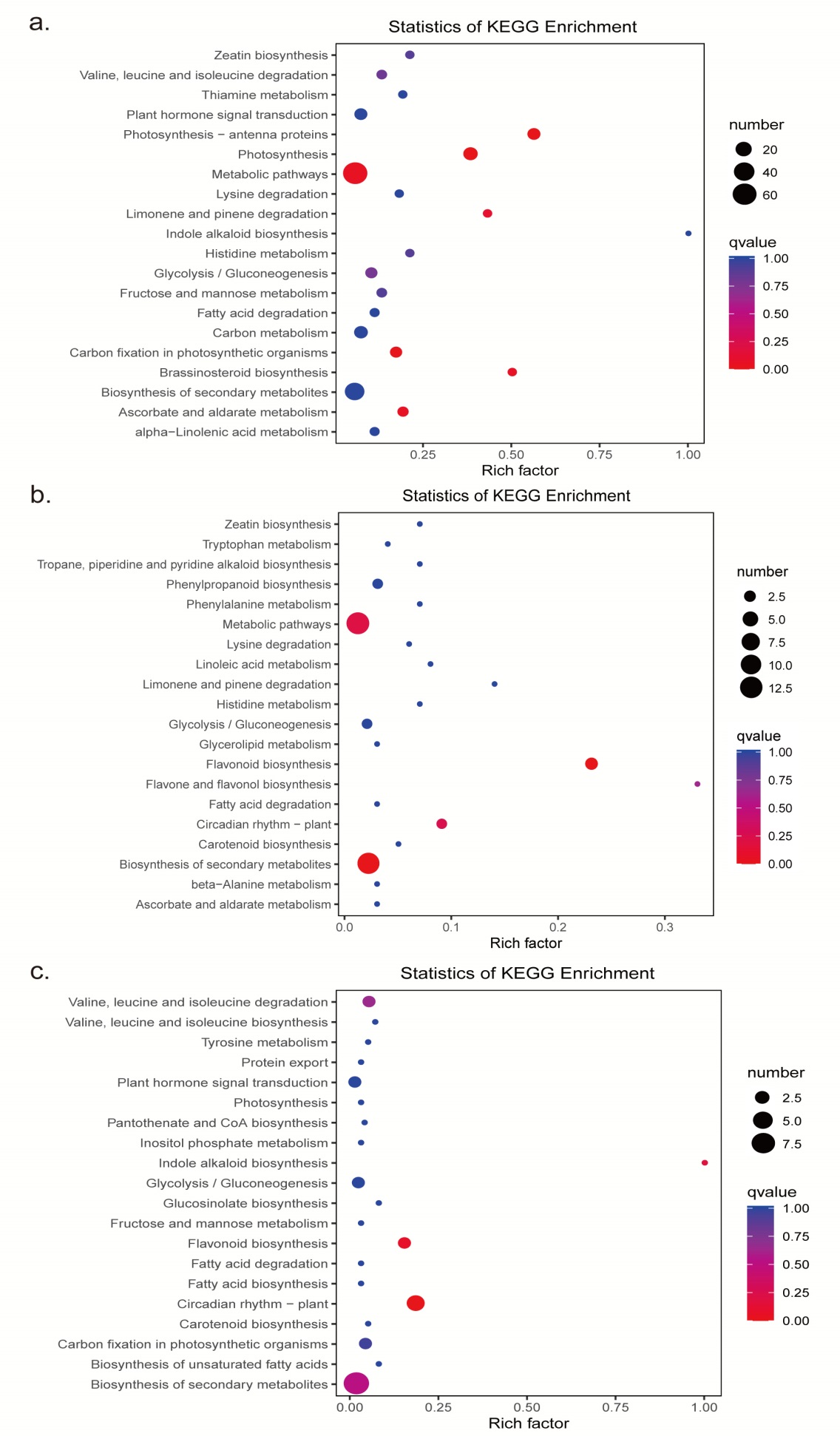


Fig. S11. Enrichment of KEGG pathways in differentially expressed genes associated with peach domestication (a), improvement (b) and differentiation (c).


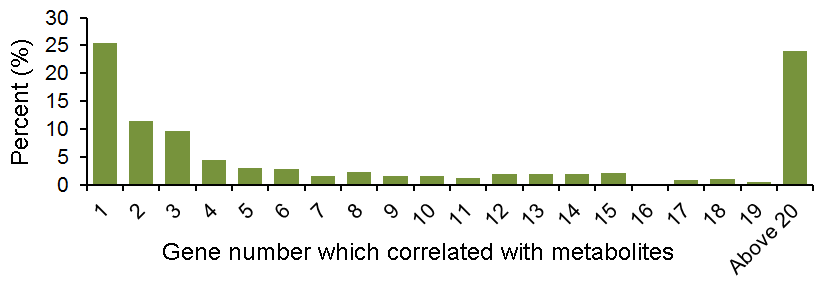


Fig. S12. Number of expressed genes correlated with the content of each metabolite.


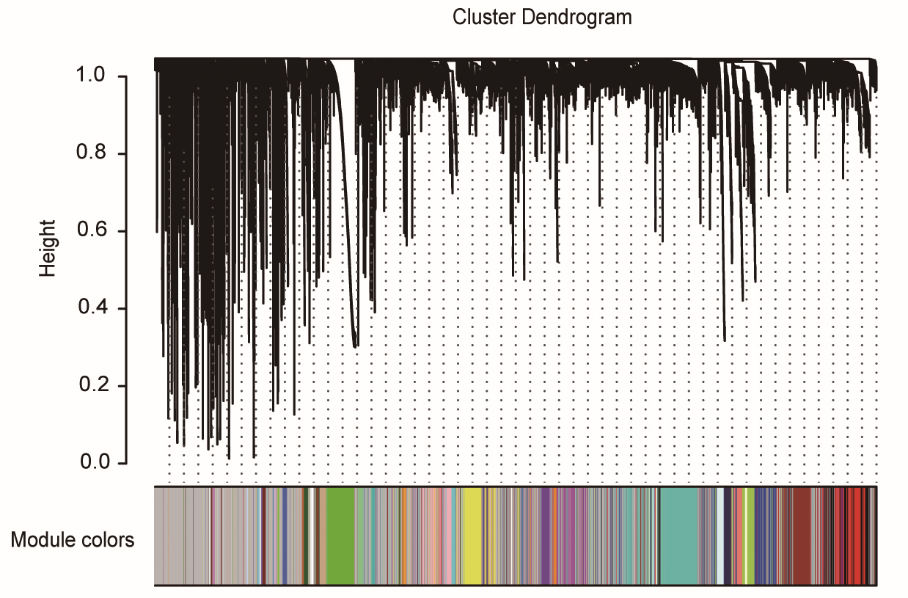


Fig. S13. Co-expression network modules constructed using weighted correlation network analysis (WGCNA) based on gene expression values. Each color indicates a different module.


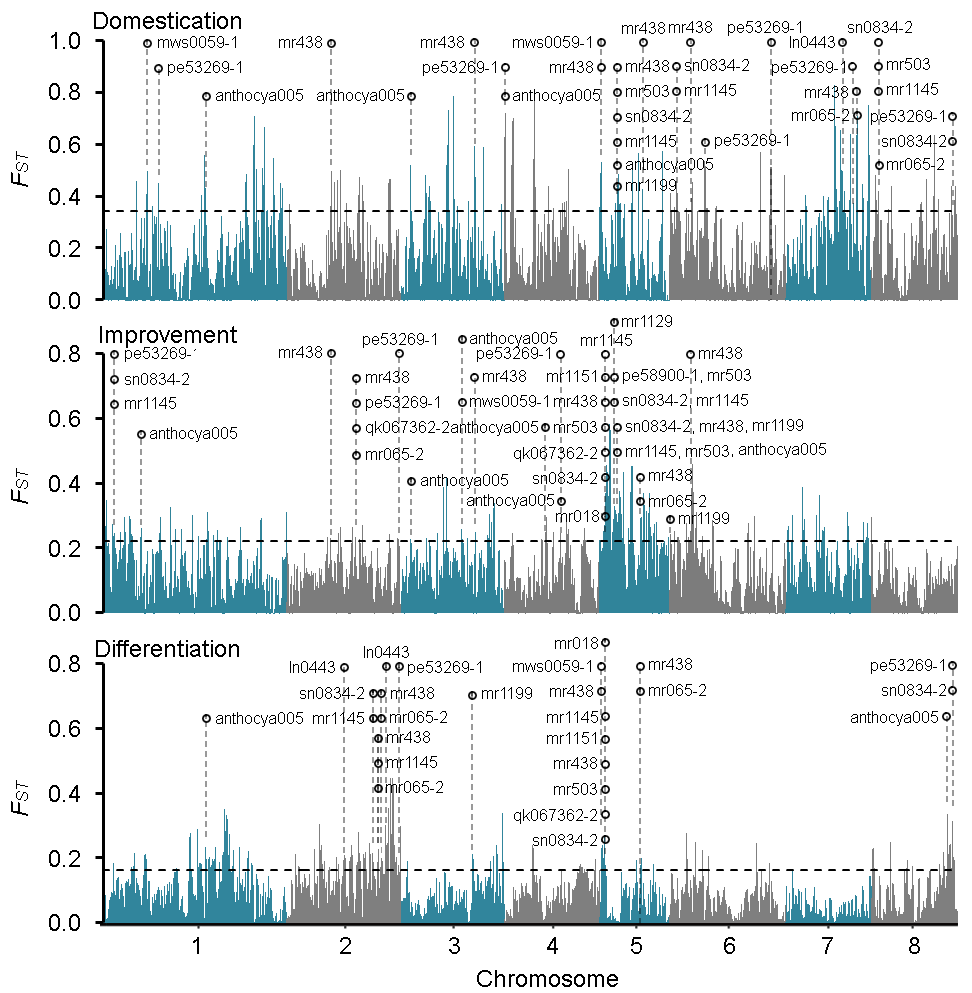


Fig. S14. Genome screening of selective sweeps in peach and their overlaps with associated SNPs of flavonoids. (a) Selective sweeps during peach domestication. (b) Selective sweeps during peach improvement. (c) Selective sweeps during differentiation between eastern and western improved varieties.


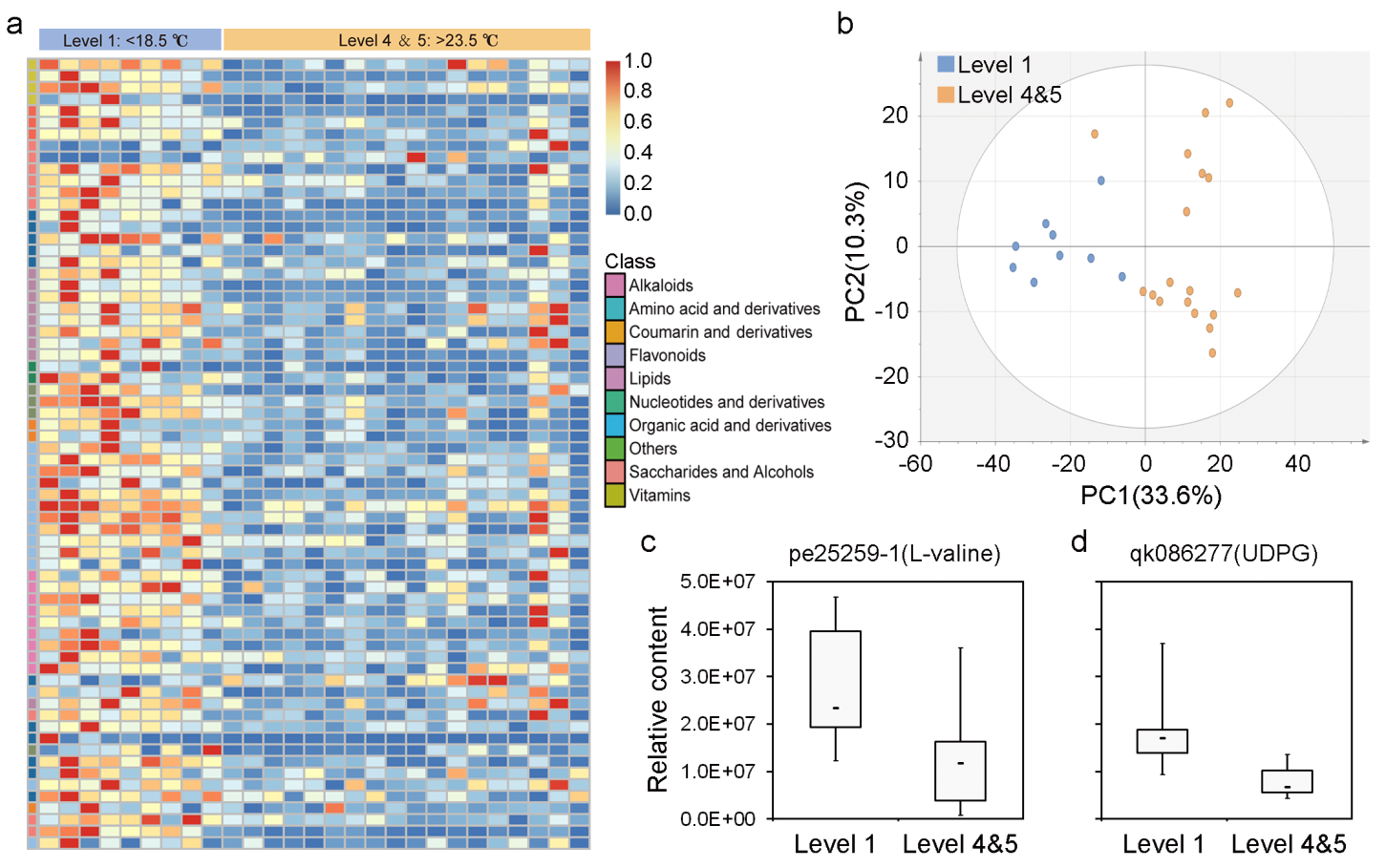


Fig. S15. Identification of differential metabolites according to temperature levels in the growing period in the origin places of peaches. (a) Heatmap of relative contents of all annotated metabolites between level 1 and level 4&5 populations of peach. (b) PCA plot of landraces according to the differential metabolites detected between level 1 and level 4&5 populations of peach. (c) Boxplot of L-valine contents in level 1 and level 4&5 populations. (d) Boxplot of UDPG contents in level 1 and level 4&5 populations.


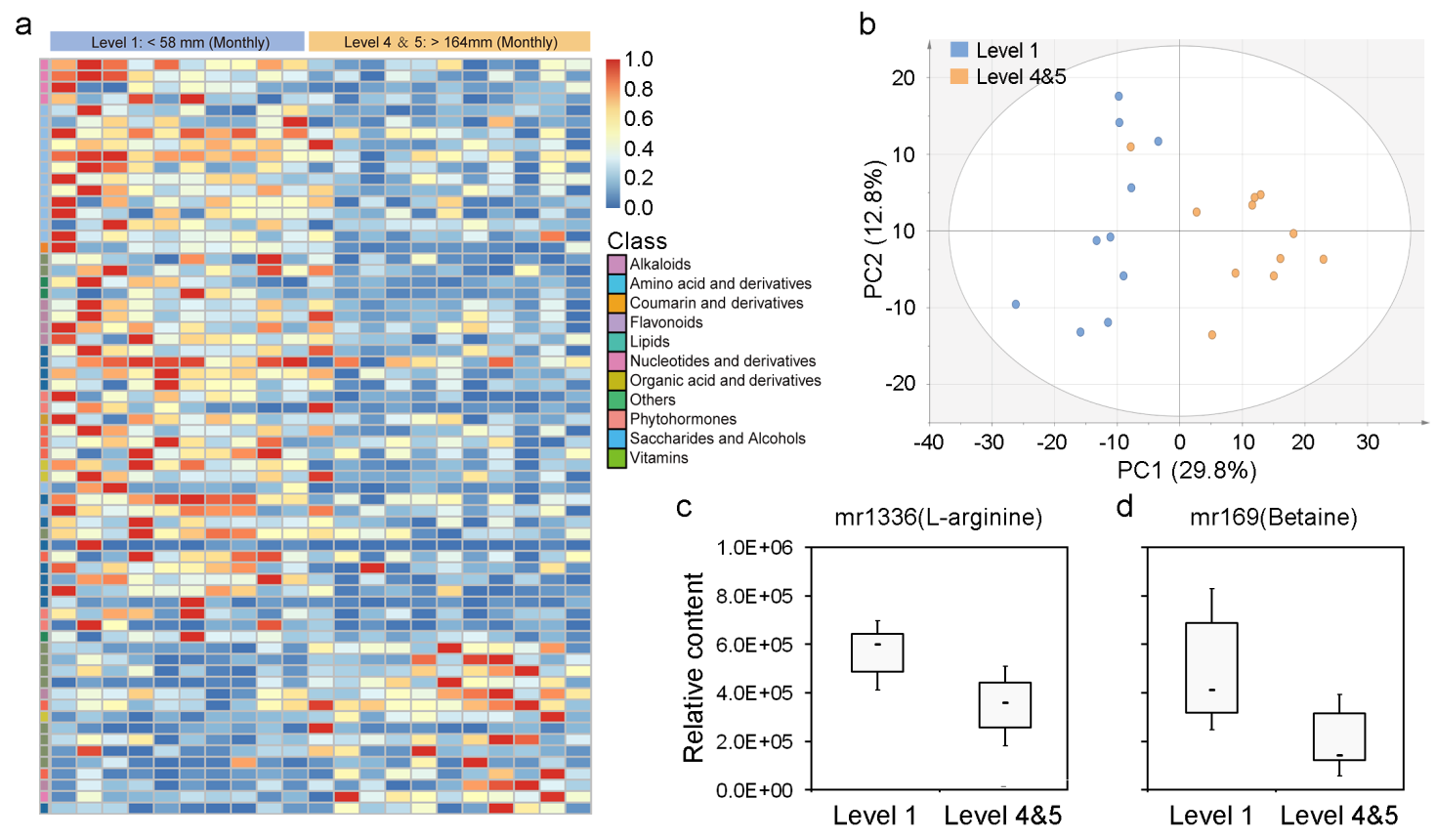
 Fig. S16. Identification of differential metabolites according to rainfall levels in the growing period in the origin places of peaches. (a) Heatmap of relative contents of all annotated metabolites between level 1 and level 4&5 populations of peach. (b) PCA plot of landraces according to the differential metabolites detected between level 1 and level 4&5 populations of peach. (c) Boxplot of L-arginine contents in level 1 and level 4&5 populations. (d) Boxplot of betaine contents in level 1 and level 4&5 populations.


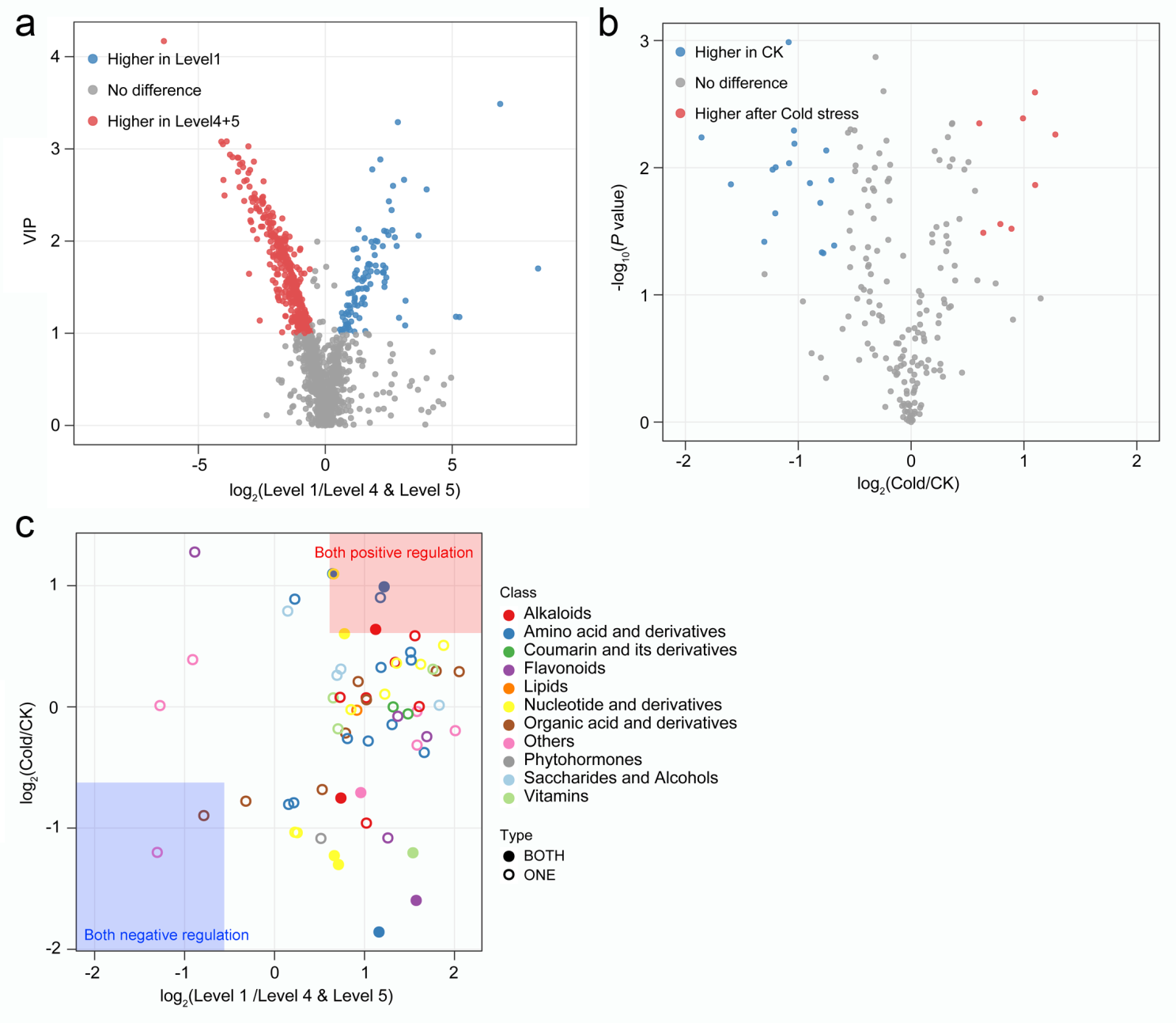


Fig. S17. Volcano plots to identify annotated metabolites responding to cold. (a) Differential metabolites identified according to different temperatures of peach origins. (b) Differential metabolites identified in fruits of peach varieties ‘Zhong Nong Jin Hui’ treated with low temperature (4 °C). (c) Differential metabolites regulated by both long and short cold inductions.


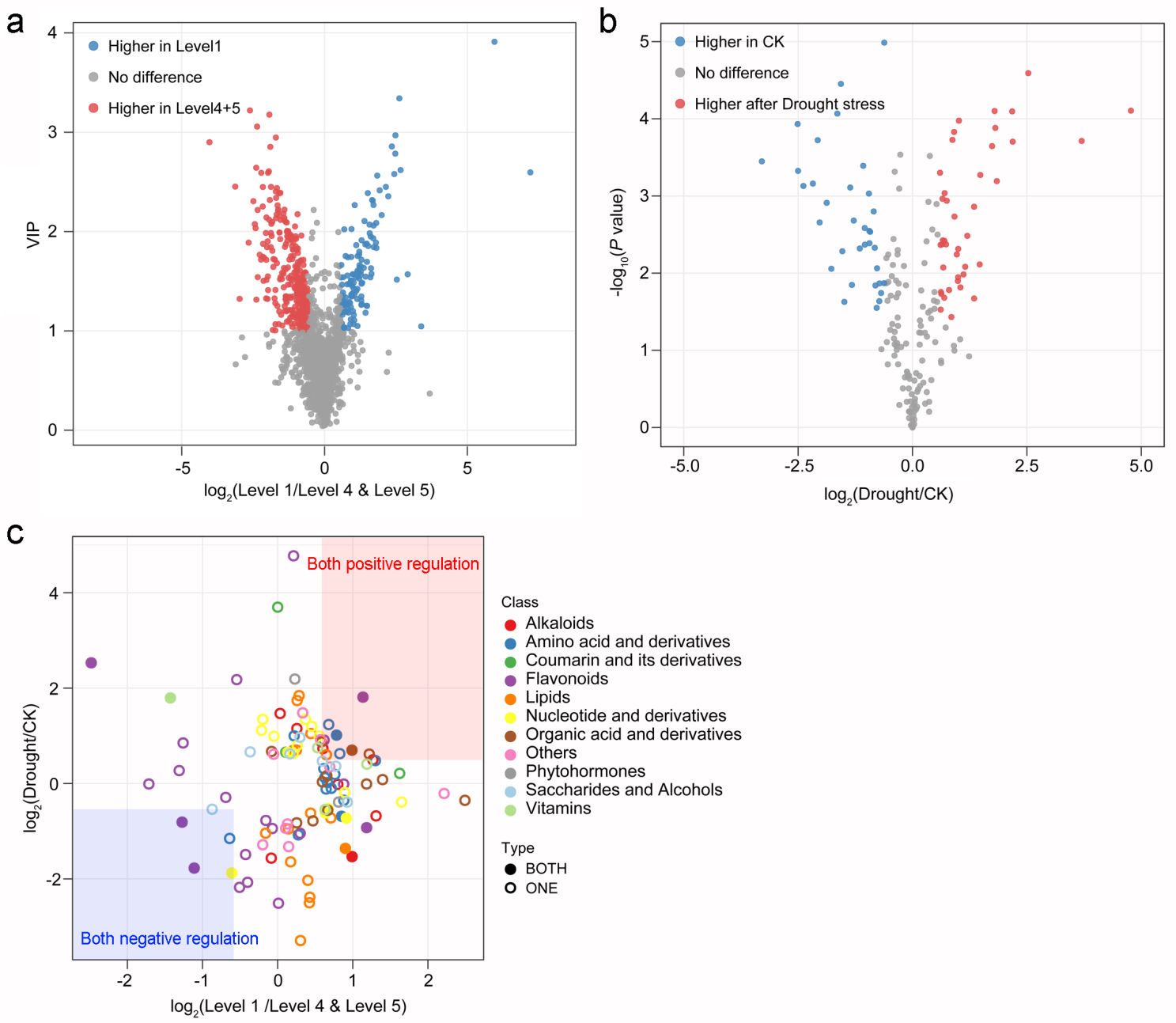


Fig. S18. Volcano plots to identify annotated metabolites responding to drought. (a) Differential metabolites identified according to different rainfalls of peach origins. (b) Differential metabolites identified in fruits of peach varieties ‘Zhong Nong Jin Hui’ treated with drought. (c) Differential metabolites regulated by both long and short drought treatments.

Fig. S19. Proline contents in leaves treated by spraying water (WT) and L-valine solution (50 mg/L) in ‘Shenzhou Li He Shui Mi’ peach trees of 15-leaves old that was induced by -4 ℃ for 24 or 72 hours.


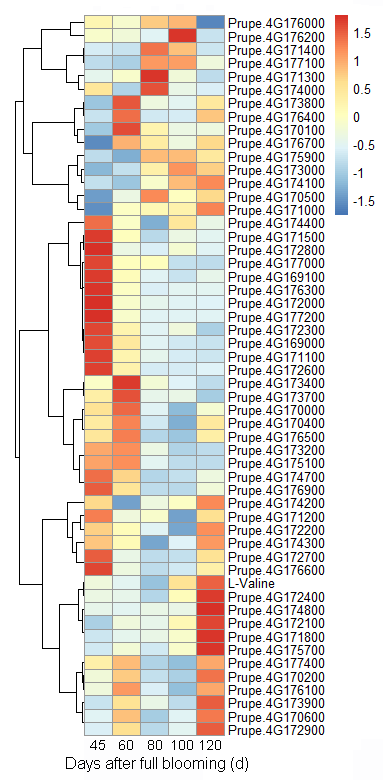


Fig. S20. L-valine contents and expression profiles of genes located in the mQTL region of L-valine on chromosome 4 during fruit development.


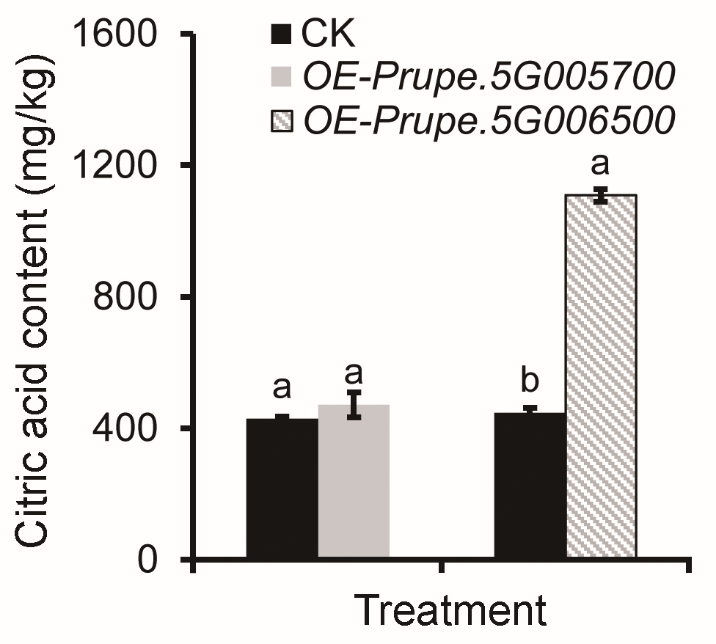


Fig. S21. Citric acid contents in lines transiently overexpressing *Prupe.5G005700* or *Prupe.5G006500* in tobacco.


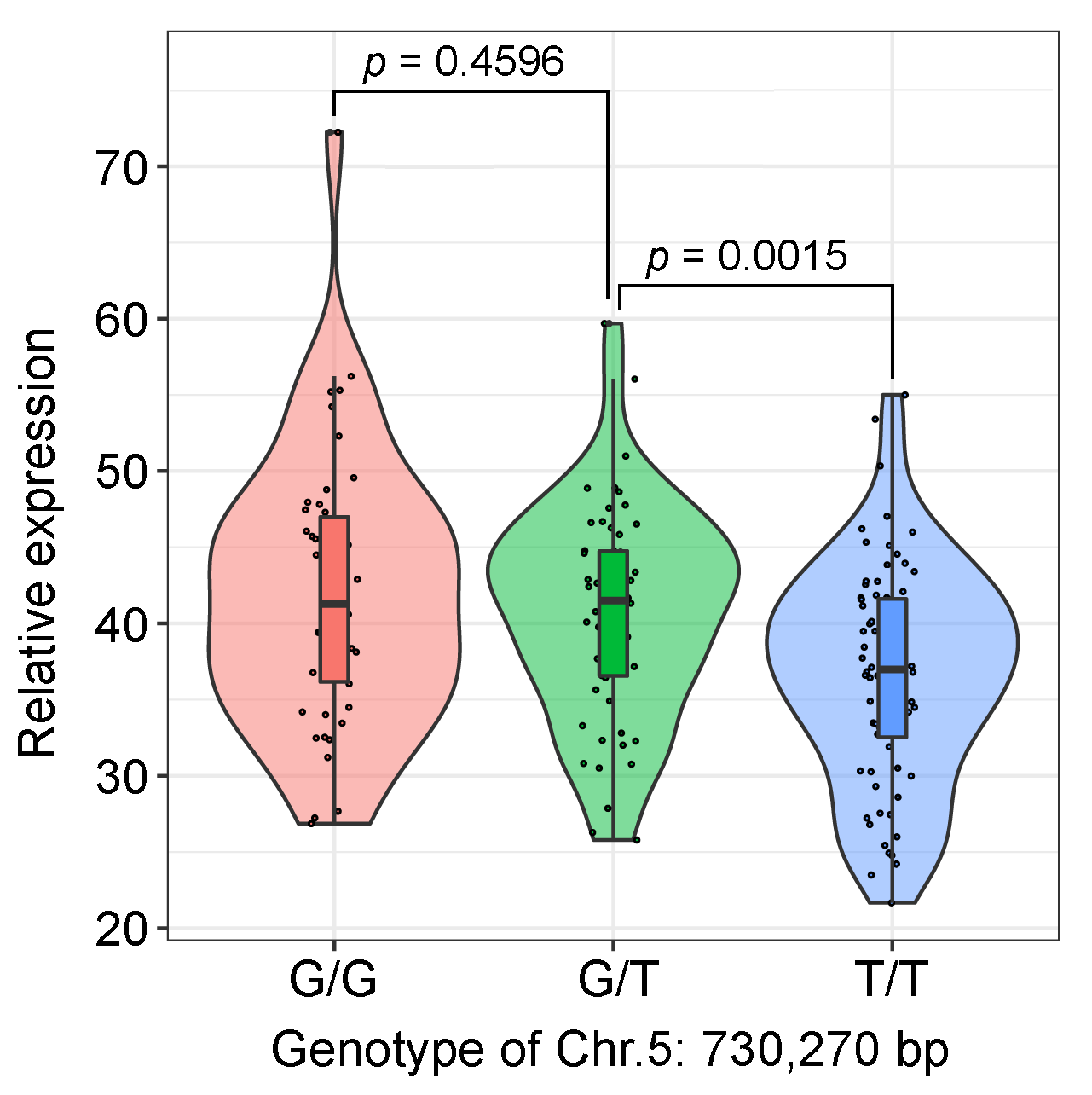


Fig. S22. Relative expression of *Prupe.5G006500* in peaches with different genotypes at position Chr. 5: 730,270 bp.


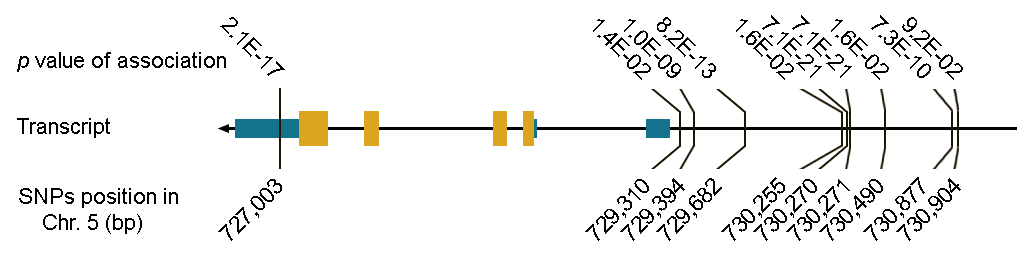


Fig. S23. Variation detection of *Prupe.5G006500* gene associated with cirtic acid contents in peach fruit. Yellow color indicates exons, and blue indicates untranslated regions; *p* values indicate significances of the associations between genotypes and critic acid contents evaluated in 252 peaches in 2016.


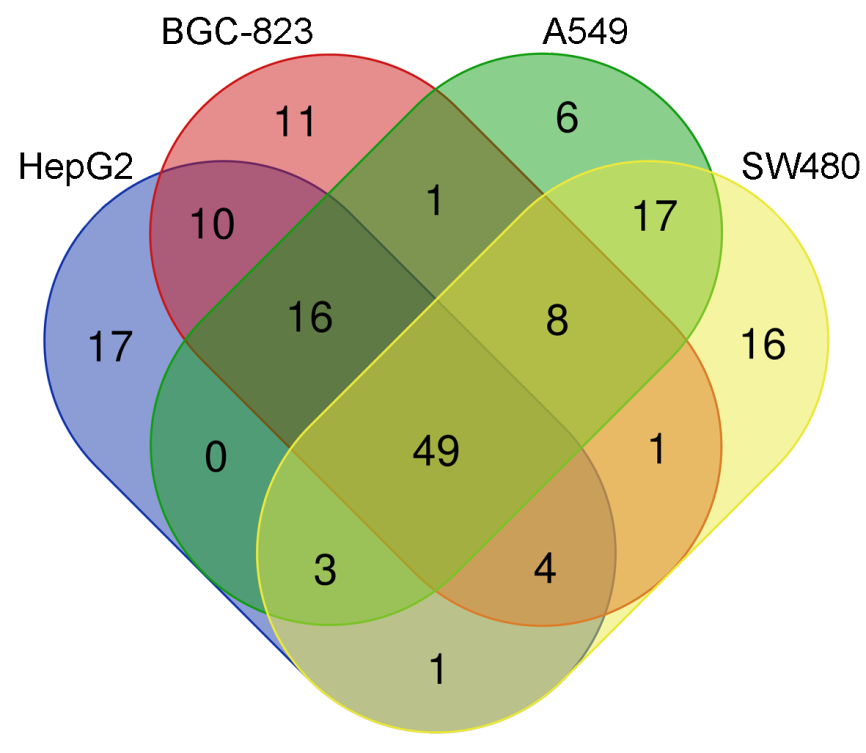


Fig. S24. Venn diagram of the top 100 metabolites with high inhibition activities to the four cancer cells.
