# Supplementary material for: Combined nature and human selections reshaped peach fruit metabolome
Source: Genome Biol. 2022 Jul 4;23:146. doi: 10.1186/s13059-022-02719-6 (PMC9254577; doi:10.1186/s13059-022-02719-6)
Supplement: Supplementary file 33 — Additional file 33. Review history. [file 13059_2022_2719_MOESM33_ESM.docx]

Review History

**First round of review**

**Reviewer 1**

**Were you able to assess all statistics in the manuscript, including the appropriateness of statistical tests used?**

No, I do not feel adequately qualified to assess the statistics.

**Comments to author:**

This is a large-scale study involving genome, transcriptome and metabolome in peach fruit. A lot of analyses have been performed and huge number of results have been generated. The comprehensive mapping of mQTL seems to have limited biological significance (only 200 metabolites are annotated, what other metabolites are, what these thousands of mQTL are and also those eQTL). I totally understand the challenge of analysis and presentation of such data. This study tried to discover some biological relevance using several examples from Fig 3 to 5. Overall, I think this is an interesting study which provides certain metabolome information for peach. I have a few comments for improving the manuscript.

Is the metabolite profiling method newly developed in this study? If not, references should be cited for metabolite extraction and LC-MS analysis. Is any standard used during extraction and analysis? This manuscript does not do a good job with clearly introducing the methods. Please check all methods and make sure information is provided.

For eQTL analysis, authors finally used only 2560 high quality SNPs identified from RNA-seq reads for analysis. The number of SNPs is very limited and the QTL mapping could be not accurate enough. In addition, authors only identified 475 cis-eQTLs, I think this is not quite normal, even these SNPs are from exons (authors define cis-eQTL within 30 kb from ATG of a gene). I believe using 486,009 SNPs for eQTL mapping is more reasonable.

Line 419, how to accurately determine 15 days before ripening? I think it is not quite possible. How to make sure these fruit samples are at similar developmental stage? Especially, fruit development of different genotypes should be very different. This is important for reliable data acquisition. When write methods, authors must describe accurately.

Line 416, should be specific about what materials are. Leaf and fruit?

Line 420, "immature fruits…. for transcriptome sequencing and eQTL analysis". Here, delete eQTL analysis.

Line 513, authors only called SNPs. Why not call InDels?

Line 571-580, transient expression methods need to cite references. Which data is from transient expression in tobacco leaf? Transient expression in peach fruits need more detailed description. More details should also be provided. For example, how L-Valine was determined for transient expressed fruit?

Figure 5c, was this done in tobacco leaf or fruit? It should be indicated in the legend. There is no method describing the measurement of these acids too. To me, OE of the candidate genes has limited effect on the level of acids. Authors should repeat and make this result is solid.

Figure 3e, the lines on right side indicate correlation between metabolites and genes? If so, each line should clearly point to the specific gene.

Line 774, it is better to define "metabolites with unknown function" as unannotated metabolites. Because the function may be still unknown even for some of the annotated metabolites.

Figure 2b, the figure legend is too simple. It is hard to understand this result. How many genes and metabolites were used to construct the network? There are only a few hundred genes and less than 100 metabolites.

In supplementary Figures 3-5, I do not see method describing how to identify differential metabolites. The threshold for determination of differential metabolites? Authors calculated the average of each metabolite in each subgroup?

Supplementary Figure 7, is it number of mQTL or number of associated SNPs? Same for Fig 2c. Authors need make sure accurately describing of SNPs and QTL.

**Reviewer 2**

**Were you able to assess all statistics in the manuscript, including the appropriateness of statistical tests used?**

Yes, and I have assessed the statistics in my report.

**Comments to author:**

In this manuscript, Cao et al. performed a multi-omics study for unraveling the genetic basis underlying several essential traits (metabolites) reshaped by nature and human selection during peach domestication and/or improvement process. This study offers a dictionary for a wealth of genomic, transcriptomic, and metabolic data, which will help the further genetic and genomic study of peach; it also increases the readers' knowledge on the biochemical basis of flavor, nutrition, and resistance. Nevertheless, I believe some issues are needed to be further addressed before the publication in Genome Biology.

1. Line 112. "We further identified the different metabolites between the two peach groups" Please clarify which "two peach groups" in this sentence? Or does this refer to "each of the above two groups"?

2. Line 114. There is a typo for "acida".

3. Line 128. The author should show more information on re-sequencing data in the main text, for example, the data size and average depth.

4. Line 137-141. Please define "hotspot" in the main text.

5. Line 113-115, Line 120-121, and Line 137-141. The manuscript has mentioned the metabolites "flavonoids and organic acids" frequently; these may have different profiles under different scenario. Thus, the current expression of "flavonoids and organic acids" seems too general. Please add more detail.

6. Line 142-144. "we collected fruits at 15 d prior to ripening of 185 accessions in 2016 for transcriptome sequencing" The author collected the samples at 15 d prior to ripening (pre-mature) for transcriptome analysis and the matured time for metabolism analysis, respectively. The reason (consideration) for this experiment design should be addressed, as in the view of most audiences, huge alternation/transition may occur in the last two weeks before fully matured.

7. Line 158. what is the criteria of the high correlation?

8. Line 171-173. "A high consistency between eQTLs (Fig. 2c) and mGWAS (Supplementary Fig. 7) on some chromosomes was found, which showed an inherent connection among genomic variation, gene expression, and metabolome." What analysis has been performed for announcing "high consistency"?

9. Line 189-191. The author should define genomic regions related to domestication or improvement in the main text instead of mentioning this vital information just in the method section.

10. Line 210. Please provide more details for "this region".

11. Line 216. what is the criteria of the moderate correlation?

12. Line 218. the location of this gene "Prupe.5G032800" should be pointed out. Why the authors highlight it?

13. Line 221. I do not understand the meaning of the higher correlation here.

14. Line 225-228. How does a transporter have the ability to regulate expressions of other genes?

15. Line 229. "Amino Acid Contributed to The Environmental Adaptability of Landraces". Although some metabolites do have functions related to environmental adaptability, the author should provide more supportive information (idea) about why the accumulation of these compounds in fruit can reflect the biochemical basis for such adaptability of peach tree.

16. Line 285-286. I think there is a mistake for which group under higher pressure.

17. Line 297-307. The authors should provide more direct evidence for the V-type ATPase gene. This gene family is reported to be associated with acid content. Therefore, it can be expected for the positive results of transient transformation experiment. Previous studies have pointed out some causal genes for low-acid locus in peach. I think the key point is that nobody could detect the causal variation in the locus. Authors have proposed that the expression of this ATPase gene had a high correlation with the citric acid content and proposed a 6-bp deletion in the intron of this gene. What is the correlation between the expression variation and deletion? Could the authors provide more evidence to explain the expression variation?

18. Line 352. The definition of ecological type or ecotype is different from landrace.

19. Line 521. missing rate < 0.8?

20. Line 545. what is the LD distance for this population? I think the window size (100 Kb) is a little bit large?

**Authors Response**

**Point-by-point responses to the reviewers’ comments:**

Reviewer #1:

This is a large-scale study involving genome, transcriptome and metabolome in peach fruit. A lot of analyses have been performed and huge number of results have been generated. The comprehensive mapping of mQTL seems to have limited biological significance (only 200 metabolites are annotated, what other metabolites are, what these thousands of mQTL are and also those eQTL). I totally understand the challenge of analysis and presentation of such data. This study tried to discover some biological relevance using several examples from Fig 3 to 5. Overall, I think this is an interesting study which provides certain metabolome information for peach. I have a few comments for improving the manuscript.

*Reply: Thank you for your comments. In the study, a total of 1858 metabolites were identified, but only 196 were annotated. I admitted that many of the results in this experiment could lose their meaning. Because the experiment was begun in several years ago, the database is indeed increasing in these subsequent years. According to your suggestion, we re-analyzed the spectrum of the unannotated metabolites in this study. At present, a total of 61 new metabolites have been identified. We have added these metabolites to the revised manuscript.*

Is the metabolite profiling method newly developed in this study? If not, references should be cited for metabolite extraction and LC-MS analysis. Is any standard used during extraction and analysis? This manuscript does not do a good job with clearly introducing the methods. Please check all methods and make sure information is provided.

*Reply: Thank you for your comments. In this study, it is not a new method for performing metabolome analysis. So, the references about metabolite extraction and LC-MS analyses (Chen et al., 2013) were added. At the same time, according to your suggestion, the method of mGWAS hotspot identification (Chen et al., 2014), fruit sampling for eQTLs analysis, the identification method of eQTLs hotspots (Silva et al., 2014), and WGCNA performing (Langfelder, P. & Horvath, 2008) were added.*

*Reference：*

*Chen, W. et al. (2013). A novel integrated method for large-scale detection, identification, and quantification of widely targeted metabolites: application in the study of rice metabolomics. Mol. Plant 6, 1769-1780.*

*Chen, W. et al. (2014). Genome-wide association analyses provide genetic and biochemical insights into natural variation in rice metabolism. Nat. Genet. 46, 714-721.*

*Langfelder, P. & Horvath, S. (2008). WGCNA: an R package for weighted correlation network analysis. BMC Bioinformatics 9, 559.*

*Silva, I. T., Rosales, R. A., Holanda, A. J., Nussenzweig, M. C. & Jankovic M. (2014) Identification of chromosomal translocation hotspots via scan statistics. Bioinformatics 30, 2551-2558.*

For eQTL analysis, authors finally used only 2560 high quality SNPs identified from RNA-seq reads for analysis. The number of SNPs is very limited and the QTL mapping could be not accurate enough. In addition, authors only identified 475 cis-eQTLs, I think this is not quite normal, even these SNPs are from exons (authors define cis-eQTL within 30 kb from ATG of a gene). I believe using 486,009 SNPs for eQTL mapping is more reasonable.

*Reply: Thank you for your comments. We also feel that using only 2,560 SNPs for eQTLs is inappropriate. However, Can we perform eQTLs analysis with 486,009 SNPs from the genome? We found that in the most of the similar studies, the SNPs called from transcriptome data were used but for genome-derived SNPs. For example, in the study of Galpaz et al. (2018), the RNA-seq data of 96 melon RILs was used to detect 82,140 SNPs. Finally, the filtering SNPs (58,328 SNPs) were used for eQTLs analysis to obtain 12,703 eQTLs. In the study of Zhang et al. (2017), the authors performed transcriptome sequencing of 240 lettuce accessions and identified 1,133,865 SNPs. Finally, using the 103,662 SNPs, eQTLs analysis was performed to obtain 5,311 eQTLs. In the study of Wang et al. (2021), a high-quality set of 974,404 SNPs was called from the RNA-seq data of pear to obtain 833,872 eQTLs putatively regulating the expression of 18,435 genes.*

*Therefore, in the revised manuscript, the SNPs identified from RNA-seq were still selected for eQTLs analysis. And in this version, we slightly reduced the thresholds for SNPs filtering. The parameters was defined as sequencing depth ≥ 3, missing rate ≥ 0.2, MAF < 0.02. Finally, a total of 75,817 SNPs were filtered. After re-performing the eQTLs analysis, the threshold of significance (P = 1.99 × 10 −12) was increased, which is significantly lower than the previous (P = 1.99 × 10 −3), resulting in a total of 5,134 cis-eQTLs and 85,899 cis-eQTLs. The results have been added in the revised manuscript.*

*Reference：*

*Galpaz, N. et al. (2018). Deciphering genetic factors that determine melon fruit-quality traits using RNA-Seq-based high-resolution QTL and eQTL mapping. Plant J. 94, 169-191.*

*Zhang, L. et al. (2017). RNA sequencing provides insights into the evolution of lettuce and the regulation of flavonoid biosynthesis. Nat. Commun. 8, 2264.*

*Wang R. Z. et al. (2021). A systems genetics approach reveals PbrNSC as a regulator of lignin and cellulose biosynthesis in stone cells of pear fruit. Genome Biol. 22, 313.*

Line 419, how to accurately determine 15 days before ripening? I think it is not quite possible. How to make sure these fruit samples are at similar developmental stage? Especially, fruit development of different genotypes should be very different. This is important for reliable data acquisition. When write methods, authors must describe accurately.

*Reply: Yes, I agree with your opinion. It is difficult to judge the maturity period of each peach. Sorry I didn't write clearly before. In fact, to determine the optimal maturity period of each variety, we estimated the maturity period of each variety based on previous experience, and then picked the fruit in 20 days before the estimated maturity date with every 5 days. The fruits were stored with liquid ammonia and measured the firmness at the same time. Then, for each variety, we drew a change pattern of fruit firmness in different ripening stages. The optimal maturity date was defined as the highest point of firmness in this year. Finally, the samples which picked in 15 days before ripening were selected and sent to the company for transcriptome sequencing. The corresponding revision has been added.*

Line 416, should be specific about what materials are. Leaf and fruit?

*Reply: Thanks, the sentence has been revised. Here, the material is the fruit.*

Line 420, "immature fruits…. for transcriptome sequencing and eQTL analysis". Here, delete eQTL analysis.

*Reply: Deleted.*

Line 513, authors only called SNPs. Why not call InDels?

*Reply: In the study, the InDels and SVs were not identified based on the following reasons. Firstly, we mainly used the SNPs of 252 peach accessions for mGWAS analysis, and we believed that the results could meet the object for localization of key metabolites. Secondly, all the 252 accessions are mainly from the two paper which published by our laboratory in 2019 (Cao et al., 2019; Li et al., 2019) and one in 2020 (Guo et al., 2020). In these articles, the authors have already performed InDels and SVs identification. Therefore, these variations were not be identified by our team again. Of course, we also thought it was helpful to perform variant detection such as InDel and SVs for identifying key variants of excellent genes. For example, in our study, Sanger sequencing was used to identify the key variants associated with L-valine and citric acid content in fruits.*

*Reference:*

*Cao, K. et al. (2019). Comparative population genomics identified genomic regions and candidate genes associated with fruit domestication traits in peach. Plant Biotechnol. J. 17, 1954-1970.*

*Li, Y. et al. (2019). Genomic analyses of an extensive collection of wild and cultivated accessions provide new insights into peach breeding history. Genome Biol. 20, 36.*

*Guo, J. et al. (2020). An integrated peach genome structural variation map uncovers genes associated with fruit traits. Genome Biol. 21, 258.*

Line 571-580, transient expression methods need to cite references. Which data is from transient expression in tobacco leaf? Transient expression in peach fruits need more detailed description. More details should also be provided. For example, how L-Valine was determined for transient expressed fruit?

*Reply: Thank you for your comments. The reference has been added in the revised manuscript, such as Zhou et al. (2016). In addition, in this study, the transient system was used in tobacco to verify the functions of key genes regulating critic acid content in flesh. Meanwhile, the system was used in peach to verify the functions of gene involved in valine and critic acid biosynthesis. Finally, the detailed meathod for evaluating L-valine and citric acid was also added.*

*Reference:*

*Zhou, H. et al. (2016). Multiple R2R3-MYB Transcription factors involved in the regulation of anthocyanin accumulation in peach flower. Front. Plant Sci. 7, 1557.*

Figure 5c, was this done in tobacco leaf or fruit? It should be indicated in the legend. There is no method describing the measurement of these acids too. To me, OE of the candidate genes has limited effect on the level of acids. Authors should repeat and make this result is solid.

*Reply: Thanks for your comments. In our study, this experiment was performed on peach fruit. And we have added it in the legend of Fig. 5. In addition, the measurement method of acid is also descripted in the experimental method of transient transformation. As your suggestion, to verify the function of this gene, we also measured the acid content of peach fruit after transient transformation using a widely targeted metabolomic technology, which also showed that the effect of Prupe.5G006500 was stronger than that of Prupe.5G005700. Since we believe that HPLC assays are more credible for specific products, the results of widely targeted metabolomic technology (as shown in Fig. 1) are not put in the original manuscript. And the other reason is that the latter is easy to lead a misunderstanding that the content of citric acid is higher than that of malic acid in peach fruit (the comparison between two metabolites cannot be performed in the widely targeted metabolomic technology).*

*Fig. 1 Citric acid and malic acid contents in lines transiently overexpressing Prupe.5G005700 or Prupe.5G006500 in peach fruits.*

Figure 3e, the lines on right side indicate correlation between metabolites and genes? If so, each line should clearly point to the specific gene.

*Reply: Thank you for your comments, the figure has been changed.*

Line 774, it is better to define "metabolites with unknown function" as unannotated metabolites. Because the function may be still unknown even for some of the annotated metabolites.

*Reply: Revised.*

Figure 2b, the figure legend is too simple. It is hard to understand this result. How many genes and metabolites were used to construct the network? There are only a few hundred genes and less than 100 metabolites.

*Reply: Thanks for your comments. The number of metabolites and genes were added to the legend. For example, in this figure, a total of 94 annotated metabolites were used. As the metabolites were annotated again, the figure was drawn with a new version.*

In supplementary Figures 3-5, I do not see method describing how to identify differential metabolites. The threshold for determination of differential metabolites? Authors calculated the average of each metabolite in each subgroup?

*Reply: Thank you for your suggestion. The method for identifying differential metabolites was added.*

In this study, the criterion which used to decide the differential metabolites was defined as Fold change (FC) ≤ 0.67 or FC ≥ 1.5, and variable importance in the projection (VIP) ≥ 1. When calculating FC, it is necessary to calculate the average value of each metabolite in each subgroup.

Supplementary Figure 7, is it number of mQTLs or number of associated SNPs? Same for Fig 2c. Authors need make sure accurately describing of SNPs and QTL.

*Reply: Thank you for your suggestion. I have checked all manuscript and found that most of them should be revised as associated SNPs.*

Reviewer #2:

In this manuscript, Cao et al. performed a multi-omics study for unraveling the genetic basis underlying several essential traits (metabolites) reshaped by nature and human selection during peach domestication and/or improvement process. This study offers a dictionary for a wealth of genomic, transcriptomic, and metabolic data, which will help the further genetic and genomic study of peach; it also increases the readers' knowledge on the biochemical basis of flavor, nutrition, and resistance. Nevertheless, I believe some issues are needed to be further addressed before the publication in Genome Biology.

1. Line 112. "We further identified the different metabolites between the two peach groups" Please clarify which "two peach groups" in this sentence? Or does this refer to "each of the above two groups"?

*Reply: Thank you for your comments. You are right. The sentence has been revised as your suggestion.*

2. Line 114. There is a typo for "acida".

*Reply: Revised.*

3. Line 128. The author should show more information on re-sequencing data in the main text, for example, the data size and average depth.

*Reply: Thank you for your suggestion. The description has been added.*

4. Line 137-141. Please define "hotspot" in the main text.

*Reply: Thank you for your suggestion. The method for hotspot identification has been added in the Methods section. For example, the distribution of associated lead SNPs along the genome was investigated by dividing the whole genome into 1 Mb partitions, and the number of lead SNPs in each segment was counted. A permutation test was used to assess the statistical significance of the deviation of the observed lead SNPs distribution per Mb from the expectation based on chance events, assuming a uniform distribution throughout the genome.*

5. Line 113-115, Line 120-121, and Line 137-141. The manuscript has mentioned the metabolites "flavonoids and organic acids" frequently; these may have different profiles under different scenario. Thus, the current expression of "flavonoids and organic acids" seems too general. Please add more detail.

*Reply: Thank you for your suggestion, I have added some detailed descriptions in the corresponding paragraph.*

6. Line 142-144. "we collected fruits at 15 d prior to ripening of 185 accessions in 2016 for transcriptome sequencing" The author collected the samples at 15 d prior to ripening (pre-mature) for transcriptome analysis and the matured time for metabolism analysis, respectively. The reason (consideration) for this experiment design should be addressed, as in the view of most audiences, huge alternation/transition may occur in the last two weeks before fully matured.

*Reply: Thank you for your comments. The reasons for sampling at different times and detailed sampling methods have been added to the revised manuscript.*

7. Line 158. what is the criteria of the high correlation?

*Reply: Sorry to make you misunderstanding. The criteria of the high correlation in line 158 is same to the above correlation, such as r > 0.4 and p < 2.23 × 10-6. I have added the criteria in the revised manuscript.*

8. Line 171-173. "A high consistency between eQTLs (Fig. 2c) and mGWAS (Supplementary Fig. 7) on some chromosomes was found, which showed an inherent connection among genomic variation, gene expression, and metabolome." What analysis has been performed for announcing "high consistency"?

*Reply: Thank you for your suggestion. We admitted that the previous description was not precise enough. In the revised manuscript, we found among hotspot of eQTLs, most of them overlapped with those hotspots of mGWAS. And the description has been added in the new edition.*

9. Line 189-191. The author should define genomic regions related to domestication or improvement in the main text instead of mentioning this vital information just in the method section.

*Reply: Thank you for your suggestion. The definitions of domestication or improvement have been added in the revised manuscript.*

10. Line 210. Please provide more details for "this region".

*Reply: Thank you for your comments. The definition of “this region” has been added.*

11. Line 216. what is the criteria of the moderate correlation?

*Reply: Thank you for your suggestion. Here, the correlation between the content change of chrysoeriol 5-O-hexoside and quercetin 3-O-glucoside and the expression of Prupe.5G006200 in peach cultivar ‘Zheng Bai 5-6’ throughout the entire fruit development process was 0.19 and 0.13. Therefore, we thought the correlation was weak. The sentence has been revised.*

12. Line 218. the location of this gene "Prupe.5G032800" should be pointed out. Why the authors highlight it?

*Reply: Thanks for your comments; the location of this gene has been added to the manuscript. It was chosen because it is on the flavonoid metabolite synthesis pathway.*

13. Line 221. I do not understand the meaning of the higher correlation here.

*Reply: Thanks for your comments. The sentence has been revised.*

14. Line 225-228. How does a transporter have the ability to regulate expressions of other genes?

*Reply: Thank you for your comments. In previous studies, the TT12 gene was reported in Arabidopsis to regulate the content of flavonoids as a transporter. Indeed, few literatures suggest that it may be involved in the biosynthesis of flavonoids as a regulatory gene. Since another expert believed that the number of SNPs used in this study was too small in the analysis of eQTLs. So, in the revised manuscript, we increased the number of SNPs and raised the threshold for eQTLs identification (P = 1.99 × 10 −12). The result showed the regulation between Prupe.5G032800 and a trans-eQTLs (Chr. 2: 3,997,543 bp) within a gene encoding a MATE efflux family protein (TT12, Prupe.2G037200) was filtering. Analysis of the reasons found that the p value of this eQTLs and Prupe.2G037200 was 1.94 × 10 −7.*

15. Line 229. "Amino Acid Contributed to The Environmental Adaptability of Landraces". Although some metabolites do have functions related to environmental adaptability, the author should provide more supportive information (idea) about why the accumulation of these compounds in fruit can reflect the biochemical basis for such adaptability of peach tree.

*Reply: Thanks for your comments. I admitted that there was no correlation between the resistance of fruit and young branches. Therefore, we sprayed some peach seedlings (P. persica cv. Shenzhou Li He Shui Mi) with 10 to 15 leaves old with 50mg/L L-valine. The result showed that the cold resistance of the whole plant was indeed enhanced by observing the increasing proline content in treatment comparing with control (spraying water). This experimental result was added in the revised manuscript. However, the L-valine in other tissues was not evaluated in the previous study. However, in terms of tissue-specific expression, the gene Prupe.4G174300 involved in L-valine biosynthesis in fruits was highly expressed in roots, phloem of branches and seeds (Fig. 2). The data was referred in our previous study (Cao et al., 2019).*

*Fig. 2 The expression of Prupe.4G174300 which involved in L-valine biosynthesis in different tissues.*

*Reference:*

*Cao, K. et al. (2019). Comparative population genomics identified genomic regions and candidate genes associated with fruit domestication traits in peach. Plant Biotechnol. J. 17, 1954-1970.*

16. Line 285-286. I think there is a mistake for which group under higher pressure.

*Reply: Thanks. Since the content of acid is relatively high in the wild species of peach, and in the bred varieties, the western group is higher than that of the eastern. Therefore, I think that the eastern group has experienced stronger human selection in this locus.*

17. Line 297-307. The authors should provide more direct evidence for the V-type ATPase gene. This gene family is reported to be associated with acid content. Therefore, it can be expected for the positive results of transient transformation experiment. Previous studies have pointed out some causal genes for low-acid locus in peach. I think the key point is that nobody could detect the causal variation in the locus. Authors have proposed that the expression of this ATPase gene had a high correlation with the citric acid content and proposed a 6-bp deletion in the intron of this gene. What is the correlation between the expression variation and deletion? Could the authors provide more evidence to explain the expression variation?

*Reply: Thank you for your comments. A 6-bp deletion was found in the intron of Prupe.5G006500 gene through Sanger sequencing in the previous study. We think it can be designed as an useful PCR marker. At the same time, we analyzed the variation of the genic and the promoter regions in the resequencing data, and found that some SNPs in promoter was related to both gene expression and citric acid content in peach fruit, providing more evidence for confirming the function of the gene. These descriptions have been added in revised manuscript as Supplementary Fig. 22 and 23.*

18. Line 352. The definition of ecological type or ecotype is different from landrace.

*Reply: Thanks for your comments. Yes, the different ecotypes are all belongs to landraces. The sentence has been revised.*

19. Line 521. missing rate < 0.8?

*Reply: Thank you for your suggestion. It is really an inappropriate description. I have revised it to “missing rate > 0.2”.*

20. Line 545. what is the LD distance for this population? I think the window size (100 Kb) is a little bit large?

*Reply: Thanks for your comment. The LD decay in peach is usually recognized as 25 kb. Therefore, the lead SNP was defined as the most significant loci in a 50 kb window (± 25kb). The lead SNPs was recalculated and counted. You can found it in Supplementary Table 8 and revised manuscript.*

**Second round of review**

**Reviewer 1**

For eQTL analysis, I understand that SNPs called from RNA-seq reads can be used for eQTL analysis. I am surprised that the detected SNP numbers and eQTL numbers increased a lot. In my previous comments, I mean that since you already have a total of 486,009 SNPs identified from re-sequenced genome (more SNPs which cover coding and non-coding regions), why not use these SNPs for eQTL analysis. I think this is more reasonable.

Heere are a few minor suggestions:

I suggest remove anti-cancer activity from keywords and include metabolome in keywords

Line 36, muti-Omics to muti-omics

Line 52-53, what does the last sentence mean? It is not accurate and clear.

Line 70, understand the genetic basis of metabolic diversity

Line 82-84, writing should be improved. the high-resolution mapping of a large number of metabolites might significantly accelerate important gene identification and pathway elucidation? The description is not correct.

Line 91, "which repeatedly detected a total of 1,858 distinct metabolite features". Are all 1,858 metabolites "repeatedly detected" in 2015 and 2016? Authors should accurately describe the results. Specific number of metabolites for each year and how many of them were both detected.

Line 219, it is wrong to say "decreased by more than 10 times". Instead, you may say decreased by 90%.

Line 133, Multi-omics Analyses to Dissect the Genetic Basis

Line 135-138, it is not necessary to describe like this. The resequencing and genomic variations have been done in previous study. You may just mention and cite the paper.

Line 142, it looks that 582 and 628 metabolites out of 1,858 metabolites have associated SNPs? The description of "More than 81.1% and 80.7% of the detected metabolites had multiple lead SNPs in 2015 and 2016" should be wrong. 81.1% and 80.7% should be referred to the ratio of 582 and 628? If so, it should not be "detected metabolites", because you detected 1,858 metabolites. It should be metabolites having significant associated SNPs. I feel many of the description is not clear and accurate, especially related to bioinformatics. I suggest authors read the whole manuscript carefully and also invite a bioinformatics expert (not student or postdoc) to read and revise.

Line 178, delete which

**Reviewer 2**

The authors have addressed most of my concerns in this revised manuscript. Thus, I think this manuscript now fulfills all the requirements for a publication in the Genome Biology journal.

**Authors Response**

**Point-by-point responses to the reviewers’ comments:**

Reviewer #1:

For eQTL analysis, I understand that SNPs called from RNA-seq reads can be used for eQTL analysis. I am surprised that the detected SNP numbers and eQTL numbers increased a lot. In my previous comments, I mean that since you already have a total of 486,009 SNPs identified from re-sequenced genome (more SNPs which cover coding and non-coding regions), why not use these SNPs for eQTL analysis. I think this is more reasonable.

*Reply: According to your comments, a total of 486,009 SNPs were used for eQTL analysis in this revision. All the results have been updated. Thanks.*

Here are a few minor suggestions:

I suggest remove anti-cancer activity from keywords and include metabolome in keywords.

*Reply: Deleted.*

Line 36, muti-Omics to muti-omics

*Reply: Revised.*

Line 52-53, what does the last sentence mean? It is not accurate and clear.

Reply: Thanks for pointing this out. This sentence has been revised.

Line 70, understand the genetic basis of metabolic diversity

*Reply: Revised.*

Line 82-84, writing should be improved. the high-resolution mapping of a large number of metabolites might significantly accelerate important gene identification and pathway elucidation? The description is not correct.

*Reply: Thank you for your comment. The sentence has been revised.*

Line 91, "which repeatedly detected a total of 1,858 distinct metabolite features". Are all 1,858 metabolites "repeatedly detected" in 2015 and 2016? Authors should accurately describe the results. Specific number of metabolites for each year and how many of them were both detected.

*Reply: We are sorry for the confusion here. In our study, a total of 70 representative samples were selected to construct a reliable metabolome library, which was then used to evaluate the 252 samples over 2 years. Therefore, the number of metabolites identified in the two years was same. We have added this to the method description section in revised manuscript to make it clear.*

Line 119, it is wrong to say "decreased by more than 10 times". Instead, you may say decreased by 90%.

*Reply: Thanks. The sentence has been revised.*

Line 133, Multi-omics Analyses to Dissect the Genetic Basis

*Reply: Revised.*

Line 135-138, it is not necessary to describe like this. The resequencing and genomic variations have been done in previous study. You may just mention and cite the paper.

*Reply: Thank you for your comments. According to your suggestion, some descriptions have been deleted. And the references were also been added.*

Line 142, it looks that 582 and 628 metabolites out of 1,858 metabolites have associated SNPs? The description of "More than 81.1% and 80.7% of the detected metabolites had multiple lead SNPs in 2015 and 2016" should be wrong. 81.1% and 80.7% should be referred to the ratio of 582 and 628? If so, it should not be "detected metabolites", because you detected 1,858 metabolites. It should be metabolites having significant associated SNPs.

*Reply: Thank you for your comment. The sentence has been revised.*

I feel many of the description is not clear and accurate, especially related to bioinformatics. I suggest authors read the whole manuscript carefully and also invite a bioinformatics expert (not student or postdoc) to read and revise.

*Reply: Thank you for your suggestion. Prof. Zhangjun Fei is an expert in plant genomics at Cornell University in the United States. Therefore, after we completed the revision, we invited him to revise this whole manuscript in detail again. Hope to further improve the professionalism of the revision.*

Line 178, delete which

*Reply: Revised.*
